# Allostery of atypical modulators at oligomeric G protein-coupled receptors

**DOI:** 10.1038/s41598-021-88399-x

**Published:** 2021-04-29

**Authors:** Rabindra V. Shivnaraine, Brendan Kelly, Gwendolynne Elmslie, Xi-Ping Huang, Yue John Dong, Margaret Seidenberg, James W. Wells, John Ellis

**Affiliations:** 1 Department of Pharmaceutical Sciences, Leslie Dan Faculty of Pharmacy, University of Toronto, 144 College Street, Toronto, ON M5S 3M2 Canada; 2 Departments of Psychiatry and Pharmacology, Hershey Medical Center, Penn State University College of Medicine, Hershey, PA 17033 USA; 3 Departments of Computer Science, Molecular and Cellular Physiology, and Structural Biology, and Institute for Computational and Mathematical Engineering, Stanford University, Stanford, CA 94305 USA; 4 Department of Psychiatry H073, Penn State University College of Medicine, 500 University Drive, Hershey, PA 17033 USA; 5 Present Address: Department of Molecular and Cellular Physiology, Stanford University School of Medicine, B163 Beckman Center, 279 Campus Drive, Stanford, CA 94305 USA; 6 Present Address: Department of Pharmacology, The National Institute of Mental Health Psychoactive Drug Screening Program (NIMH PDSP), University of North Carolina at Chapel Hill, Chapel Hill, NC 27599 USA

**Keywords:** Biochemistry, Chemical biology, Computational biology and bioinformatics, Drug discovery

## Abstract

Many G protein-coupled receptors (GPCRs) are therapeutic targets, with most drugs acting at the orthosteric site. Some GPCRs also possess allosteric sites, which have become a focus of drug discovery. In the M_2_ muscarinic receptor, allosteric modulators regulate the binding and functional effects of orthosteric ligands through a mix of conformational changes, steric hindrance and electrostatic repulsion transmitted within and between the constituent protomers of an oligomer. Tacrine has been called an atypical modulator because it exhibits positive cooperativity, as revealed by Hill coefficients greater than 1 in its negative allosteric effect on binding and response. Radioligand binding and molecular dynamics simulations were used to probe the mechanism of that modulation in monomers and oligomers of wild-type and mutant M_2_ receptors. Tacrine is not atypical at monomers, which indicates that its atypical effects are a property of the receptor in its oligomeric state. These results illustrate that oligomerization of the M_2_ receptor has functional consequences.

## Introduction

G protein-coupled receptors (GPCRs) regulate cellular signaling through conformational changes and effects on protein–protein interactions^1^. According to the traditional view, a monomeric receptor undergoes an agonist-induced transition from an inactive state to an active state^2^. The activated GPCR then couples to intracellular proteins in a sequence that begins with G proteins, followed in turn by G Protein-coupled Receptor Kinases (GRKs) and Arrestins^3^. This canonical process is consistent with much biophysical data, and it has informed much activity in the realm of drug discovery^4^. It is a working hypothesis, however, and more recent developments have necessitated refinements and extensions.

GPCRs now are known to be multi-conformational and to act directly on different effectors depending upon the conformation favored by the agonist, an effect known as *biased signaling*^3^. They also are known to occur as oligomers, allowing for protein–protein interactions between neighboring receptors as well as between the receptor and other proteins in the signaling pathway^5–7^. Finally, some GPCRs possess allosteric sites that are topographically distinct from the orthosteric site and allow for the modulation of signaling by allosteric ligands^7^.

GPCRs are localized at the cell membrane within highly compartmentalized domains known as ‘hot spots,’ as has been observed by single-particle tracking at low average levels of receptor expression^8^. Measurements of Total Internal Reflection Fluorescence (TIRF) at more physiologic levels of expression have shown that M_1_ muscarinic receptors labeled with Cy3B-telenzepine can occur as a mixture of monomers and dimers^9^, although such estimates of oligomeric size can be confounded by the power density of the exciting laser^6^. Tracking of eGFP- and mCherry-containing constructs by Fluorescence Correlation Spectroscopy (FCS), which monitors single particles within regions of ~ 500 nm in diameter, has shown that oligomers of the M_2_ muscarinic receptor can interact with G proteins^5^. Localization and concentration of the receptor may favor oligomerization and account for the fast kinetics of signaling, raising the possibility that regulation of the local density serves as a tuning mechanism in receptor-mediated signaling^10^.

Oligomers appear to underlie the effects of allosteric ligands at the M_2_ muscarinic receptor^7^. Allosteric modulation of the M_2_ receptor by small molecules appears to involve a blend of steric hindrance, conformational stabilization and electrostatic interactions^11^. It typically is seen as an intramolecular effect within mutually independent monomers, but that view has become too narrow. Modulators such as gallamine and strychnine can exhibit complex, multiphasic binding patterns that are indicative of heterogeneity within the population of receptors. Such complexity is not consistent with the notion of a monomeric receptor, nor is it observed in preparations of purified monomers; rather, it appears to arise from cooperative interactions between the protomers of an oligomeric array^7^. This is analogous to the observed role of oligomers of the M_2_ receptor in the characteristic allosteric effects of guanylyl nucleotides and G proteins^12^.

Atypical allosteric modulators are so named because they exhibit binding curves with Hill coefficients that are significantly greater than 1 and sometimes approach 2^13^. Although such an effect is indicative of positive cooperativity, the underlying mechanism is not understood. It has been postulated to result from two molecules of the modulator binding within the extracellular vestibule of one monomeric receptor^14^, but the existence of oligomers suggests other possibilities. To clarify the relationship between atypical behavior and the multiple sites of an oligomer, we have examined the interaction between the M_2_ muscarinic receptor and the atypical modulator tacrine (tetrahydroaminoacridine, or THA).

Oligomers and purified monomers of the receptor were compared for the effect of tacrine on the binding of the orthosteric antagonists *N*-[^3^H]methylscopolamine (NMS) and [^3^H]quinuclidinylbenzilate (QNB). Allosteric effects characteristic of atypical modulators such as tacrine were observed in preparations of oligomers but not in preparations of monomers. The possible location of allosteric binding sites at the extracellular surface of the receptor was explored in molecular dynamics simulations, and the identified sites were confirmed by site-directed mutagenesis and subsequent binding studies. Taken together, our results indicate that the allosteric effects commonly observed with atypical modulators arise, like those of gallamine and strychnine, from a mix of cooperative interactions within and between the protomers of an oligomer. The general ability of GPCRs to form oligomers suggests that such complexity may be a common feature of allosteric modulation within this class of receptors.

## Results

### Effect of tacrine on the binding profiles of [^3^H]QNB and [^3^H]NMS

**Figure 1:**
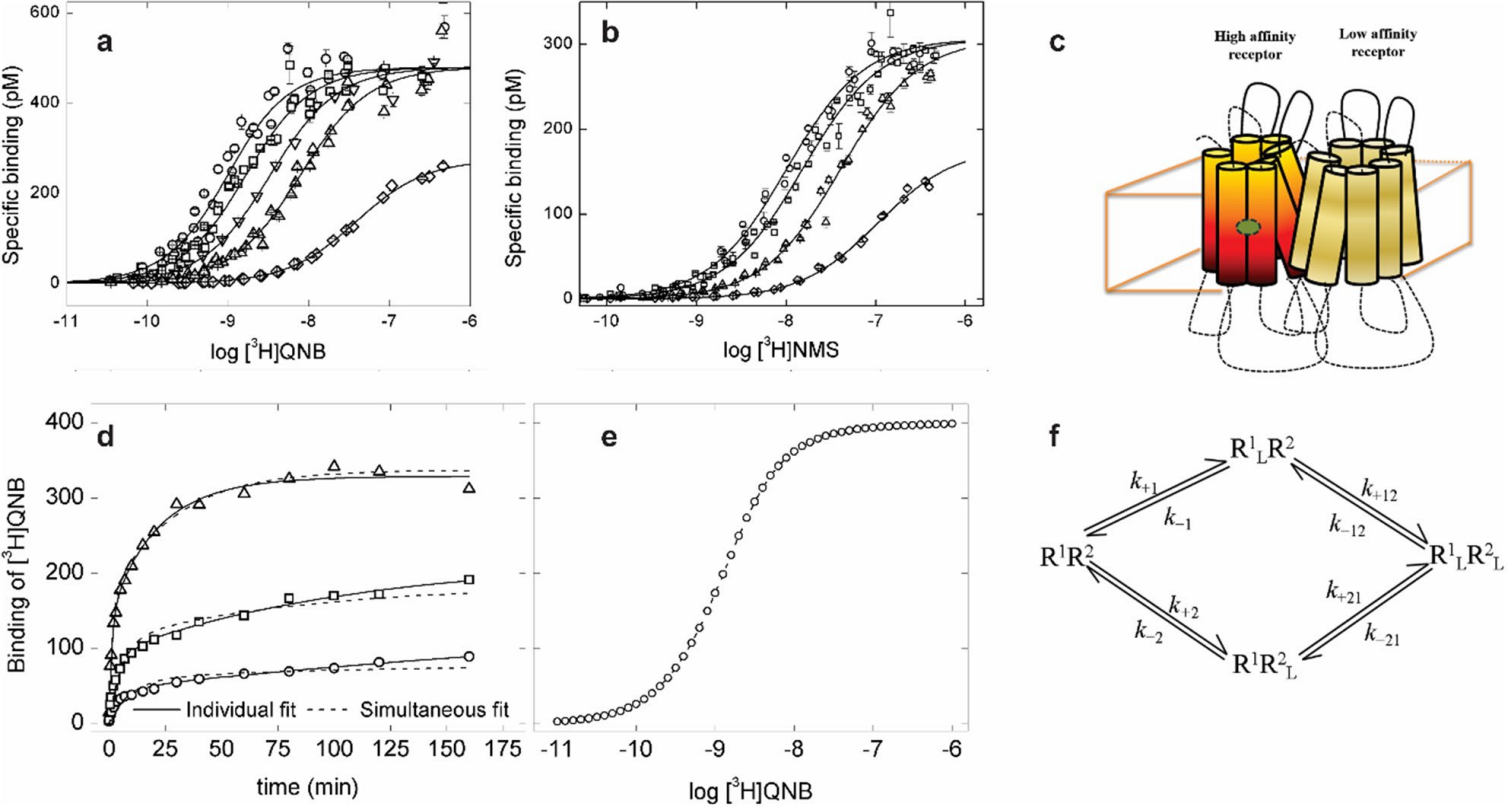
Intermolecular effects in the interaction of orthosteric ligands and tacrine with solubilized M_2_ receptor from *Sf*9 cells. (**a**,**b**) Binding at equilibrium. The receptor was mixed with [^3^H]QNB (**a**) or [^3^H]NMS (**b**) taken alone (open circle) or together with tacrine at the following concentrations: 10 µM (open square), 100 µM (open upside down triangle), 0.32 mM (open triangle), and 3.2 mM (open rhombus). Binding was measured following equilibration of the samples at 30 °C for 30 h ([^3^H]QNB) or 15 h ([^3^H]NMS). The lines depict the best fit of Eq. (2) (*n* = 1), and the parametric values are listed in Table S1. (**c**) A graphic depiction of interactions within the dimeric receptor described by Scheme 1. (**d**) Association of [^3^H]QNB. Receptor was mixed with [^3^H]QNB (open circle, 0.32 nM; open square, 1.0 nM; open triangle, 5.6 nM), and the mixture was incubated at 30 °C for the times shown on the abscissa. Three experiments were carried out at each concentration of the radioligand. Data represented in the figure were combined with data on the dissociation of [^3^H]QNB from receptor pretreated with the radioligand at a concentration of 1 nM (*N* = 3), and the complete set was analyzed according to Scheme 1. Single values of the rate constants and the co-operativity factor *α* were common to all of the data. The total concentration of receptor ([R]_t_) was measured independently and fixed at 400 pM. Three such analyses included only those data acquired at the same concentration [^3^H]QNB, and the fitted curves are shown as solid lines; a fourth analysis included all of the data, and the fitted curves are shown as dotted lines. The parametric values from all analyses are listed in Table S2. Further details are described in Figure S2. (**e**) Predicted binding of [^3^H]QNB at equilibrium. The line was simulated according to Scheme 1, taking the parametric values shown in Table S2 (Analysis 4) and integrating until the system attained equilibrium. An analysis of the simulated curve according to Eq. (2) (*n* = 1) gave the following: log *K* =  − 8.91 and *n*_H_ = 1.01. (**f**) Scheme 1. A dimeric receptor (R^1^R^2^) binds a ligand (L) at one orthosteric site (R^1^_L_R^2^, R^1^R^2^_L_) or both (R^1^_L_R^2^_L_). Further details are described in “Methods” and Supplementary Information.

The M_2_ muscarinic receptor has been shown to exist at least partly as an oligomer, most likely a tetramer^7,15^. One functional consequence of that arrangement is cooperativity in the binding of orthosteric and allosteric ligands to their respective sites within the complex^7^. Another is an apparent difference in the binding capacity of the receptor for different ligands under some conditions^16^. Differences in capacity also can be seen in Fig. 1, which shows the binding of [^3^H]QNB (Fig. 1a) and [^3^H]NMS (Fig. 1b) to M_2_ receptor extracted from *Sf*9 membranes and equilibrated with and without the allosteric modulator tacrine.

At any concentration of tacrine, the apparent capacity for [^3^H]QNB exceeded that for [^3^H]NMS by about 55% (*B*_max_, Table S1). The discrepancy suggests that some of the orthosteric sites were of anomalously weak affinity for [^3^H]NMS^16,17^. At high concentrations of tacrine, the apparent capacity for both radioligands was reduced by 42% (*B*_max_, Table S1). This latter reduction is inconsistent with the notion of a monomeric receptor with only two sites. In such a system at equilibrium, the capacity for [^3^H]NMS would be unaffected by tacrine irrespective of whether it acts via the allosteric site (Scheme S2A, Equation S7 in the Supplementary Information) or by competing with the radioligand for the orthosteric site (Scheme S2C, Equation S9). The attainment of equilibrium was ensured by incubation of the samples for up to 21 h, indicating that the reduction in capacity was not a kinetic artifact.

Tacrine also reduced the apparent affinity of the receptor for [^3^H]QNB and [^3^H]NMS (*K*, Table S1). The effect taken alone—and therefore disregarding the change in *B*_max_—is in quantitative agreement with that expected of a monomer (Scheme S2). Substituting the measured values of *K* listed in Table S1 for *K*_app_ in Equation S8 returns a value of 33 μM for the dissociation constant of tacrine at the allosteric site of an otherwise vacant receptor (*K*_A_); the corresponding values of *α* are 110 and 14 for [^3^H]QNB and [^3^H]NMS, respectively (log *α*  = 2.04 ± 0.23 and 1.15 ± 0.13).

### Kinetics of binding of [^3^H]QNB and [^3^H]NMS

**Scheme 1:**
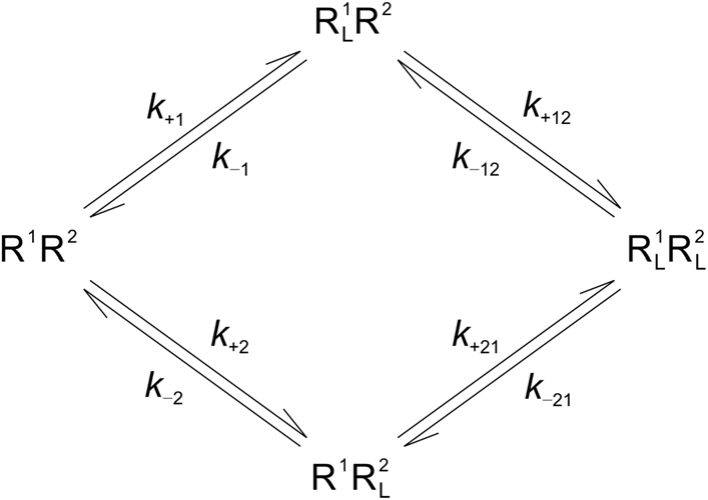
Homotropic Cooperativity in the Time-dependent Binding of a Ligand to the Two Sites of a Dimeric Receptor.

In an oligomer, differences in apparent capacity could arise from differences in the affinity of vacant protomers for the ligand (asymmetry), co-operative interactions between successive equivalents of the ligand^15^, or both (Fig. 1c). To explore these possibilities, we examined the binding kinetics of [^3^H]QNB (Fig. 1d) and [^3^H]NMS (Fig. S3) at different concentrations of each radioligand. The data were analyzed in terms of a scheme in which R^1^ and R^2^ are the constituent protomers of a potentially asymmetric and cooperative dimer (Fig. 1f, Scheme 1). Oligomers of GPCRs often have been viewed as dimers^18^, and a dimer is the simplest oligomer for practical analytical purposes. When dealing with mathematically formulated models of cooperativity, the number of parameters quickly becomes intractable as the number of constituent protomers is increased beyond two. Analyses in terms of a dimer therefore were used as a comparatively simple but practical way in which to probe the ability of an oligomer to account for the data. Details regarding the formulation and use of the model are described in “Methods” and the Supplementary Information (Section 1).

The association of [^3^H]QNB with M_2_ receptor extracted from *Sf*9 cells was followed at three concentrations of the radioligand (i.e., 0.32 nM, 1.0 nM, and 5.6 nM) (Fig. 1d), and the time-course was bi-exponential in each case (Eq. 1a, *n* = 2, *P* < 0.001). The corresponding time-course of dissociation was mono-exponential at each concentration as described previously^19^ (data not shown), and the three rate constants were essentially the same (*k*_obsd_, Eq. 1b). The data for association and dissociation were analyzed simultaneously in terms of Scheme 1, with those at different concentrations of [^3^H]QNB taken separately and together. The fitted curves are depicted by the lines in Fig. 1d, and the parametric values are listed in Table S2. The contribution of each species to total specific binding at 1 nM [^3^H]QNB is illustrated in Figure S2.

Whereas Scheme 1 can describe the association of [^3^H]QNB at any one concentration of the radioligand (Fig. 1d, solid lines), there are small but appreciable deviations when the parameters are shared by all of the data in a mechanistically consistent manner (Fig. 1d, dotted lines). Although the model appears to be inadequate, it provides at least a first approximation of the data; moreover, the binding profile computed for a system at equilibrium (Fig. 1e, log *K* =  − 8.91) compares favorably with that obtained when equilibrium was attained in the binding assay (Fig. 1a and Table S1, log *K* =  − 9.18). The fitted parametric values from Scheme 1 indicate that the system is both asymmetric (*K*_1_ ≠ *K*_2_) and co-operative (*α* < 1) (Table S2), at least when interpreted as a dimer. It appears that these two effects offset each other to yield a Hill coefficient of 1 when the simulation is continued until the system approaches equilibrium (Fig. 1e).

The association of [^3^H]NMS with the receptor exhibited a pronounced overshoot in which specific binding at each concentration of the radioligand increased to a maximum at about 20 min and decreased thereafter (Fig. S3a). Each trace approached an asymptote greater than zero, and the levels of binding at the longest time of incubation are in good agreement with the corresponding points on a binding profile measured after equilibration for 15 h (Fig. S3b). Both the affinity and the Hill coefficient of the latter are typical of digitonin-solubilized M_2_ receptor from *Sf*9 membranes (log *K* =  − 8.02 ± 0.02, *n*_H_ = 1.01 ± 0.01). The time-dependent decrease in binding therefore appears to derive from a redistribution of sites from states of higher to lower affinity and not from an irreversible process such as thermal inactivation.

An overshoot in the binding of a radioligand could arise in principle from negative co-operativity between ligands binding at neighboring protomers (Fig. S4). In the case of [^3^H]NMS and Scheme 1, however, the model cannot reconcile the magnitude of the initial increase with that of the subsequent decline (Fig. S3a, Table S4). The constraint lies in the single rate of dissociation, which forces the model to assign all effects to differences in the rates of association. Such effects can be accommodated more readily when both rate constants can vary (Fig. S3, Table S4). With the restriction on the rates of dissociation, however, more than two interacting sites are required to achieve the level of binding that precedes equilibration in the case of [^3^H]NMS. It follows that the receptor must be larger than a dimer if the data are to be described in terms of interactions within an oligomer.

### Effects of tacrine on the kinetics of binding

We have shown previously that cooperative effects indicative of an oligomer are revealed by allosteric ligands in binding studies conducted at equilibrium^7^. Such effects also are evident with tacrine, which was examined in studies on the kinetics of binding and at equilibrium. In digitonin-solubilized preparations from *Sf*9 cells, tacrine acted to slow the association of [^3^H]QNB (Fig. S5) and [^3^H]NMS (Fig. S6). Whereas the time-course of the reaction was at least biexponential with either radioligand taken alone (Figs. 1 and S3), it was mono-exponential at even the lowest concentration of tacrine. This shift to simpler kinetics included elimination of the marked decline in the binding of [^3^H]NMS at longer times (cf. Fig. S3a and Fig. S6). It also was independent of the level of occupancy by the orthosteric ligand, at least in the case of [^3^H]QNB (Fig. S5 and Table S5). Like the overshoot observed in the absence of tacrine, it can be rationalized in terms of interactions within an oligomer (Fig. S7).

Tacrine also slowed the dissociation of [^3^H]QNB (data not shown) and [^3^H]NMS (Fig. S6), confirming the allosteric nature of its effect on the solubilized receptor. The time-course was mono-exponential with each radioligand at all concentrations tacrine (Fig. S6A–C), and the rate constants for [^3^H]NMS are listed in Table S5.

To confirm the atypical nature of the interaction between tacrine and a liganded receptor^13^, the rate of dissociation of [^3^H]NMS was measured at graded concentrations of the allosteric ligand in membranes and digitonin-solubilized extracts from porcine atria and *Sf*9 cells (Fig. S8). Fitted estimates of the rate constant [*k*_obsd_, Eq. (1b)] were normalized to that in the absence of tacrine (*k*_0_), and the dependence of the ratio (*k*_obsd_/*k*_0_) on the concentration of tacrine was analyzed according to Eq. (3). The data and fitted curves are shown in Fig. S8, and the parametric values are listed in Table S6. The Hill coefficient for the effect of the allosteric ligand on the dissociation of [^3^H]NMS exceeded 1 in all preparations, suggesting that tacrine acted via at least two allosteric sites to which it bound in a positively cooperative manner.

### Equilibration of solubilized M_2_ receptor with tacrine and [^3^H]NMS

**Figure 2:**
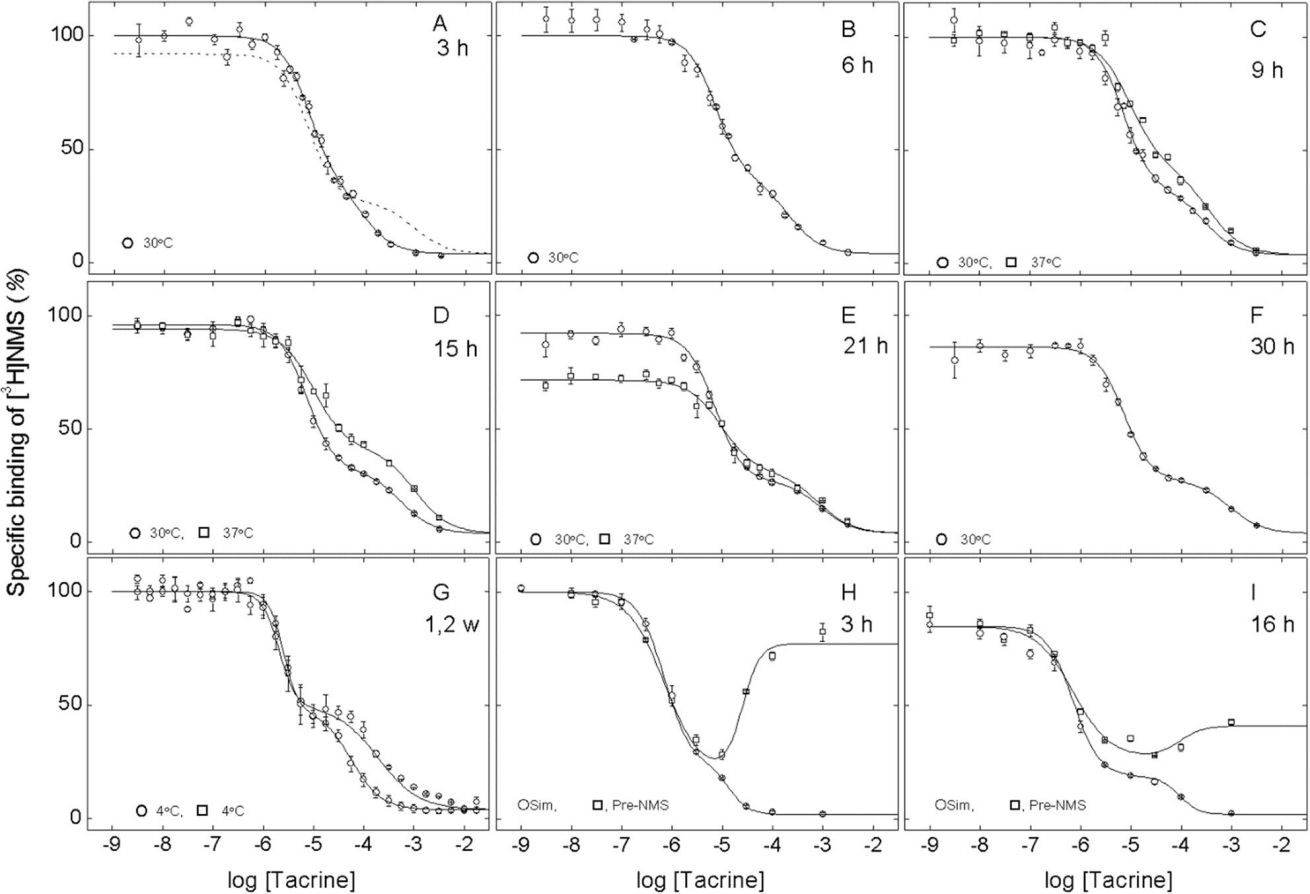
Equilibration of [^3^H]NMS and tacrine with solubilized M_2_ receptor from porcine atria. (**A**–**G**) Simultaneous addition of the two ligands. Aliquots of extract were incubated at 4 °C (open circle, open rhombus, **G**), 30 °C (open circle, **A**–**F**), or 37 °C (open square, **C–E**) with [^3^H]NMS (10 nM) plus tacrine at the concentrations shown on the abscissa. Binding was measured after incubation of the reaction mixture for different times as follows: 3 h (**A**), 6 h (**B**), 9 h (**C**), 15 h (**D**), 21 h (**E**), 30 h (**F**), 1 week (open circle, **G**), and 2 weeks (open rhombus, **G**). The solid lines depict the best fits of Eq. (3) (*n* = 2) to all of the data acquired at the same temperature taken together, and the fitted parametric values are listed in Table S7. The fitted curve in **F** is reproduced as the broken line in **A**. (**H**,**I**) Sequential addition of [^3^H]NMS and tacrine to receptor in membranes from *Sf*9 cells (open square). Receptor-containing membranes were incubated with [^3^H]NMS (0.3 nM) for 30 min at 24 ºC; tacrine then was added at the concentrations shown on the abscissa, and binding was measured after further incubation for 3 h (**H**) and 16 h (**I**) at 24 °C. As a control, membranes also were mixed simultaneously with [^3^H]NMS and tacrine (open circle), and binding was measured after incubation for 3 h (**H**) (filled circle) and 16 h (**I**) (filled square) at 24 °C. The lines depict the best fit of Eq. (3) (*n* = 2) to data obtained after sequential or simultaneous addition of the two ligands, and the parametric values are listed in Table S8. Details regarding the normalization of the data in all panels are described in “Methods”.

The atypical nature of the interaction with tacrine was examined further in studies at equilibrium. To identify the conditions necessary to achieve equilibrium, the binding pattern was measured at three temperatures (i.e., 4 °C, 30 °C, and 37 °C, Fig. 2) and after periods of incubation from 3 h to 2 weeks. Because the rate of equilibration also is affected by the order of mixing^19^, binding was compared after the simultaneous and sequential addition of the two ligands.

When tacrine and [^3^H]NMS were added simultaneously to M_2_ receptor extracted from porcine atria, tacrine was inhibitory at all temperatures and at all concentrations of the radioligand. The binding profile at 30 °C was biphasic under all conditions but broadened as the time of incubation increased from 3 to 30 h (Fig. 2A–F, Table S7). An empirical description of the data in terms of Eq. (3) (*n* = 2) indicates that the broadening derived from a tenfold increase in the value of *K*_*j*_ at the sites of weaker affinity, from 0.09 mM to a limiting value of 0.9 mM (Table S7). There was no appreciable or consistent change in *K*_*j*_ at the sites of higher affinity (*K*_1_ = 7.2 μM), in the Hill coefficient for either class of sites (*n*_H(1)_ = 1.60, *n*_H(2)_ = 1.36), or in the apparent distribution of sites between the two classes (*F*_2_ = 0.30). Essentially the same result was obtained at 37 °C (Fig. 2C–E) and 4 °C (Fig. 2G) except that equilibration was more rapid at the higher temperature. Similar results were obtained with M_2_ receptor extracted from *Sf*9 cells (Fig. S9, Table S7).

Receptor in extracts from *Sf*9 cells was stable for at least 21 h at 30 °C, in that binding in the absence of tacrine was unchanged between 9 and 21 h (Fig. S9). Receptor extracted from porcine atria was stable for at least 2 weeks at 4 °C (Fig. 2G). The atrial preparation was stable for up to 9 h at 30 °C in the absence of tacrine (Fig. 2A–C), but further incubation led to a decrease of about 20% after 30 h (Fig. 2D–F). Upon incubation at 37 °C, binding decreased by about 30% after 21 h in the absence of tacrine (Fig. 2E). The Hill coefficients associated with both classes of sites generally were independent of time and exceeded 1, most notably in the case of *n*_H(1)_ (Table S7). It follows that higher values of *n*_H_, which imply positive co-operativity in the binding of tacrine, were not an artifact of instability or inactivation of the receptor over the course of the incubation.

The order of mixing was examined in assays conducted at 24 °C on M_2_ receptor in membranes from *Sf*9 cells (Fig. 2H,I; Table S8). Samples were incubated for 3 h and 16 h. The results after the simultaneous addition of tacrine and [^3^H]NMS resembled those described above for solubilized preparations. The binding profile was biphasic downward, and prolonged incubation was accompanied by an increase in *K*_2_ with little or no change in other parameters of Eq. (3) (*n* = 2) (Table S8). In contrast, pre-treatment of the receptor with [^3^H]NMS resulted in a U-shaped binding profile. The fitted values of *K*_*j*_ and *n*_H(*j*)_ from Eq. (3) (*n* = 2) are similar to those obtained when the two ligands were added together, and the value of *K*_2_ increased over time. In addition, the asymptotic level of binding at saturating concentrations of tacrine decreased by about 50% between 3 and 16 h. The binding profile therefore appeared to be converging upon that obtained when tacrine and [^3^H]NMS were added together, presumably as the system equilibrated. Further incubation was precluded by a time-dependent loss of receptor that is evident in a concomitant but smaller decrease in the level of binding in the absence of tacrine.

### The nature of biphasic inhibition by tacrine

**Figure 3:**
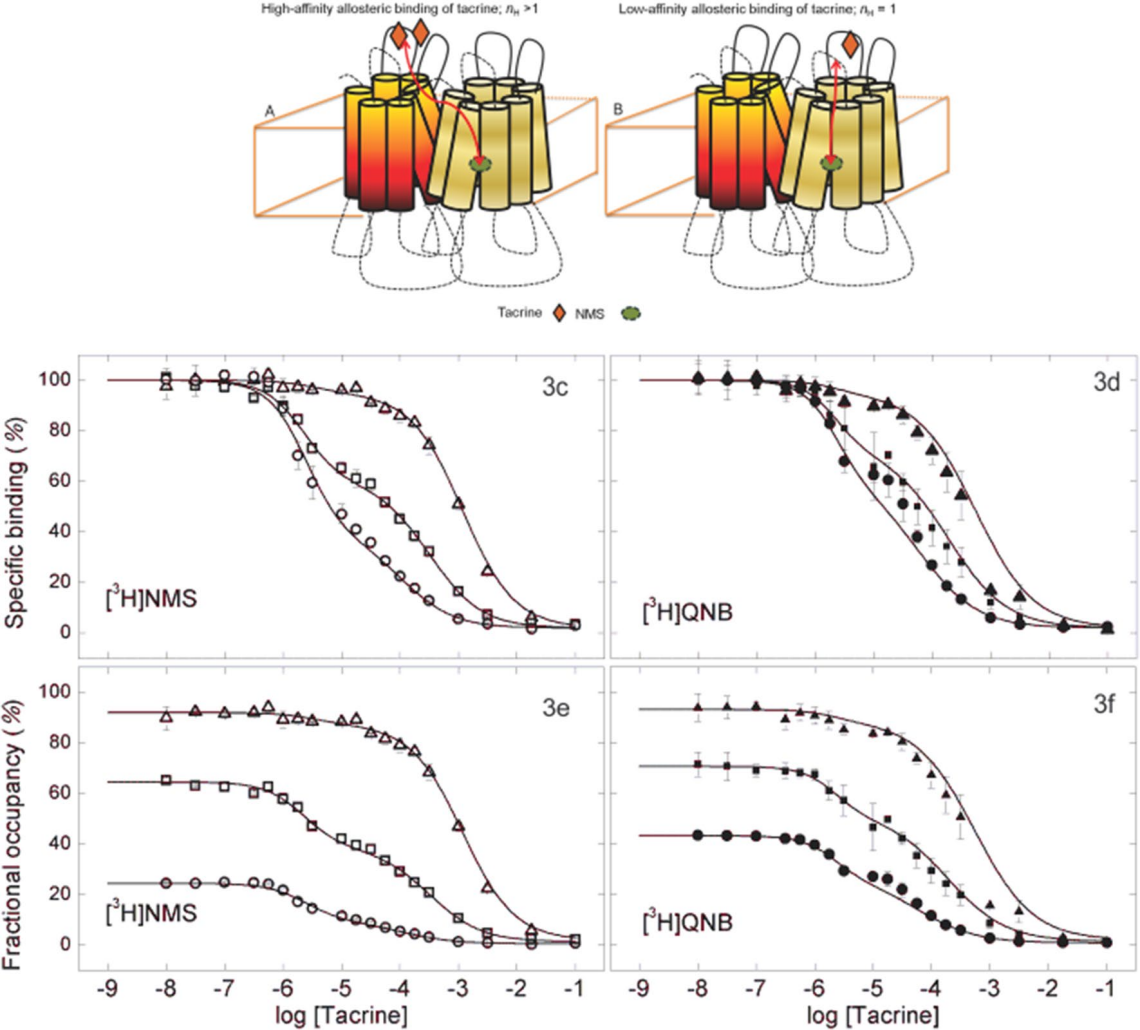
Effect of the concentration of [^3^H]NMS and [^3^H]QNB on the binding of tacrine to solubilized M_2_ receptor from *Sf*9 cells. (**A**,**B**) A schematic representation of inter- and intra-molecular allosteric modulation within an oligomer, shown here as a dimer, one protomer of which is occupied by NMS. Two equivalents of tacrine bind with high affinity and in a cooperative manner to the protomer with a vacant orthosteric site (left panel). One equivalent of tacrine binds with low affinity to the protomer with NMS at the orthosteric site (right panel). In each case, tacrine acts as a negative allosteric modulator of NMS. Tacrine and either [^3^H]NMS (**C**,**E**) or [^3^H]QNB (**D**,**F**) were mixed simultaneously with receptor, and the solution was equilibrated for 21 h ([^3^H]NMS) or 40 h ([^3^H]QNB) at 30 °C. The concentrations of each radioligand and the corresponding levels of occupancy in the absence of tacrine were as follows: for [^3^H]NMS, 3.16 nM and 24.4% (open circle), 17.8 nM and 64.5% (open square), 112 nM and 92% (open triangle); for [^3^H]QNB, 1.01 nM and 57.4% (filled circle), 1.79 nM and 70.6% (filled square), 10 nM and 93% (filled triangle). Three experiments were performed at each concentration. The data represented in the figure plus similar data acquired at three additional concentrations of [^3^H]NMS or [^3^H]QNB were analyzed simultaneously in terms of Eq. (3) (*n* = 2). The fitted curves are depicted by the lines in the figure, and the parametric values are listed in Table S9.

Changes in occupancy of the orthosteric site have been shown to shift the fractions of sites exhibiting high and low affinity for the allosteric ligand, an effect that offers some insight into the underlying interactions^19^. M_2_ receptor extracted from *Sf*9 membranes therefore was examined for the inhibitory effect of tacrine at six concentrations of [^3^H]NMS and [^3^H]QNB. The fraction of liganded receptors in the absence of tacrine ranged from 24 to 92% in the case of [^3^H]NMS (3.16–112 nM) and from 30 to 93% in the case of [^3^H]QNB (0.316–10.0 nM). All the binding profiles were biphasic downward (Fig. 3C–F), and the data were analyzed according to Eq. (3) (*n* = 2) (Table S9).

An increase in the concentration of either radioligand was accompanied by an increase in the fraction of sites ostensibly of low affinity for tacrine (*F*_2_, Table S9); at the highest concentration, virtually all of the sites were of low affinity. The effect on *F*_2_ was accompanied by a small increase in *K*_1_, but there was no consistent change in *K*_2_ or in either value of *n*_H(*j*)_. The value of *n*_H(1)_ exceeded 1 with both radioligands, although the difference was significant only with [^3^H]NMS; *n*_H(2)_ was indistinguishable from 1 in each case.

The effects illustrated in Fig. 3C–F suggest an interpretation based on a model described previously and illustrated in Fig. 3A^7^. The observed increase in the fitted value of *F*_2_ tracks the fraction of receptors occupied by [^3^H]NMS or [^3^H]QNB in the absence of tacrine. Liganded receptors therefore appear to be of lower affinity for tacrine, whereas receptors with a vacant orthosteric site are of higher affinity. It follows that the inhibitory effect of tacrine acting at the allosteric sites of lower affinity is intramolecular in nature, and the effect at the sites of higher affinity is intermolecular. Also, the Hill coefficient for tacrine typically exceeded 1 at the sites of higher affinity and was indistinguishable from 1 at the sites of lower affinity. Intermolecular effects therefore appear to involve two or more molecules of tacrine (Fig. 3A), whereas an intramolecular effect appears to involve only one (Fig. 3B).

### Effects of tacrine at purified monomers of the M_2_ receptor

**Figure 4:**
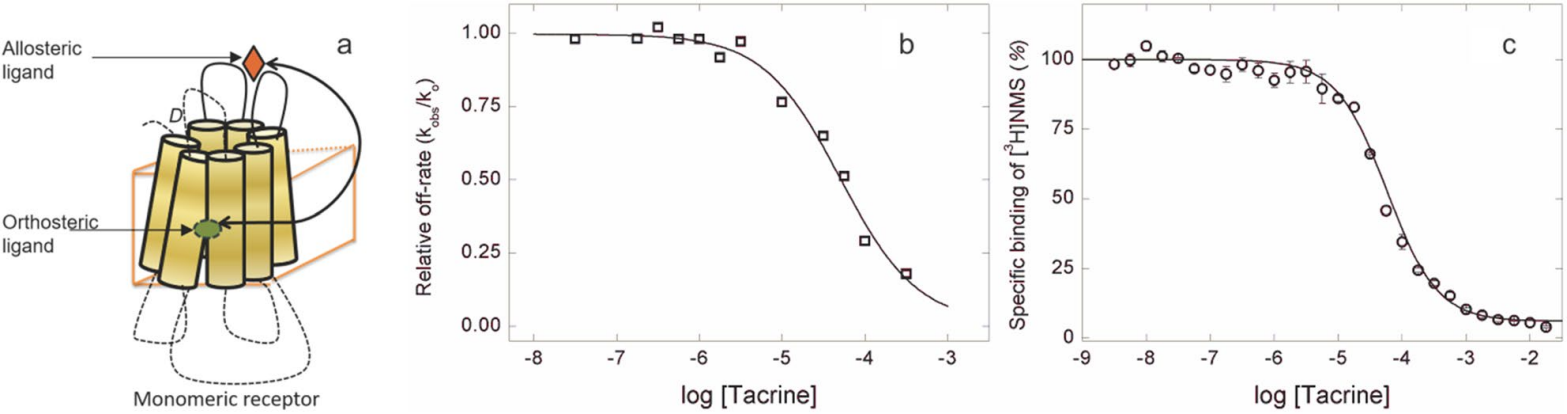
Effect of tacrine on the rate of dissociation and equilibrium binding of [^3^H]NMS at monomers of the M_2_ receptor purified from *Sf*9 cells. (**a**) A graphic depiction of the interaction between allosteric and orthosteric ligands within a monomeric receptor. (**b**) Purified monomers were labelled with [^3^H]NMS (10 nM), and the rate constant of dissociation was measured for the radioligand alone (*k*_0_) and together with tacrine (*k*_obsd_). The normalized rate constants (*k*_obsd_/*k*_0_) at graded concentrations of tacrine are shown in the figure, where the line depicts the best fit of Eq. (3) (*n* = 1). The parametric values are as follows: log *K* =  − 4.30 ± 0.13 and *n*_H_ = 0.94 ± 0.19. (**c**) Purified monomers were mixed simultaneously with [^3^H]NMS (10 nM) and tacrine at the concentrations shown on the abscissa, and binding was measured following incubation for 21 h at 30 °C. The data from 4 experiments were analyzed in terms of Eq. (3) (*n* = 1), and the fitted curve is shown in the figure. The parametric values are as follows: log *K* =  − 4.24 ± 0.02 and *n*_H_ = 1.09 ± 0.04.

Allosteric effects within a monomeric receptor will be exclusively intramolecular (Fig. 4a). The data and accompanying model in Fig. 3 suggest, therefore, that a monomer will resemble an oligomer with a high level of occupancy at the orthosteric site. To test this prediction, monomers of the M_2_ receptor were prepared from *Sf*9 membranes^7^ and examined for the effect of tacrine on the dissociation kinetics and equilibrium binding of [^3^H]NMS at 30 °C. The oligomeric status of the preparation was confirmed by densitometric analyses of western blots^7,15^, which indicated that 84 ± 5% of the cross-linked receptor migrated as a monomer (*N* = 4). The balance migrated as a mixture of oligomers.

Tacrine slowed the dissociation of [^3^H]NMS from purified monomers, and the time-course of the reaction was mono-exponential at all concentrations of the allosteric ligand. The data were analyzed in terms of Eq. (1b) (*n* = 1), and the normalized estimates of the rate constant (*k*_obsd_/*k*_0_) at graded concentrations of tacrine are plotted in Fig. 4b. The inhibitory effect of tacrine on the binding of [^3^H]NMS after equilibration of the samples for 21 h is plotted in Fig. 4c. In each case, the Hill coefficient for the dose-dependent effect of tacrine is indistinguishable from 1 (Eq. 3, *n* = 1). Purified monomers of the M_2_ receptor therefore were affected by tacrine in the manner expected for a homogeneous population of mutually independent allosteric sites, in marked contrast to the more complex behavior observed with the receptor in native membranes from porcine atria and heterologous cell lines and in unprocessed solubilized extracts.

### Molecular dynamics of tacrine binding to the M_2_ receptor

Allosteric effects during and after the attainment of equilibrium suggest that there are two sites for tacrine on an otherwise vacant receptor but only one site in the presence of an orthosteric ligand. To probe the structural basis for such an arrangement, we performed molecular dynamics simulations on the binding of tacrine to a monomeric M_2_ muscarinic receptor with and without NMS at the orthosteric site.

**Figure 5:**
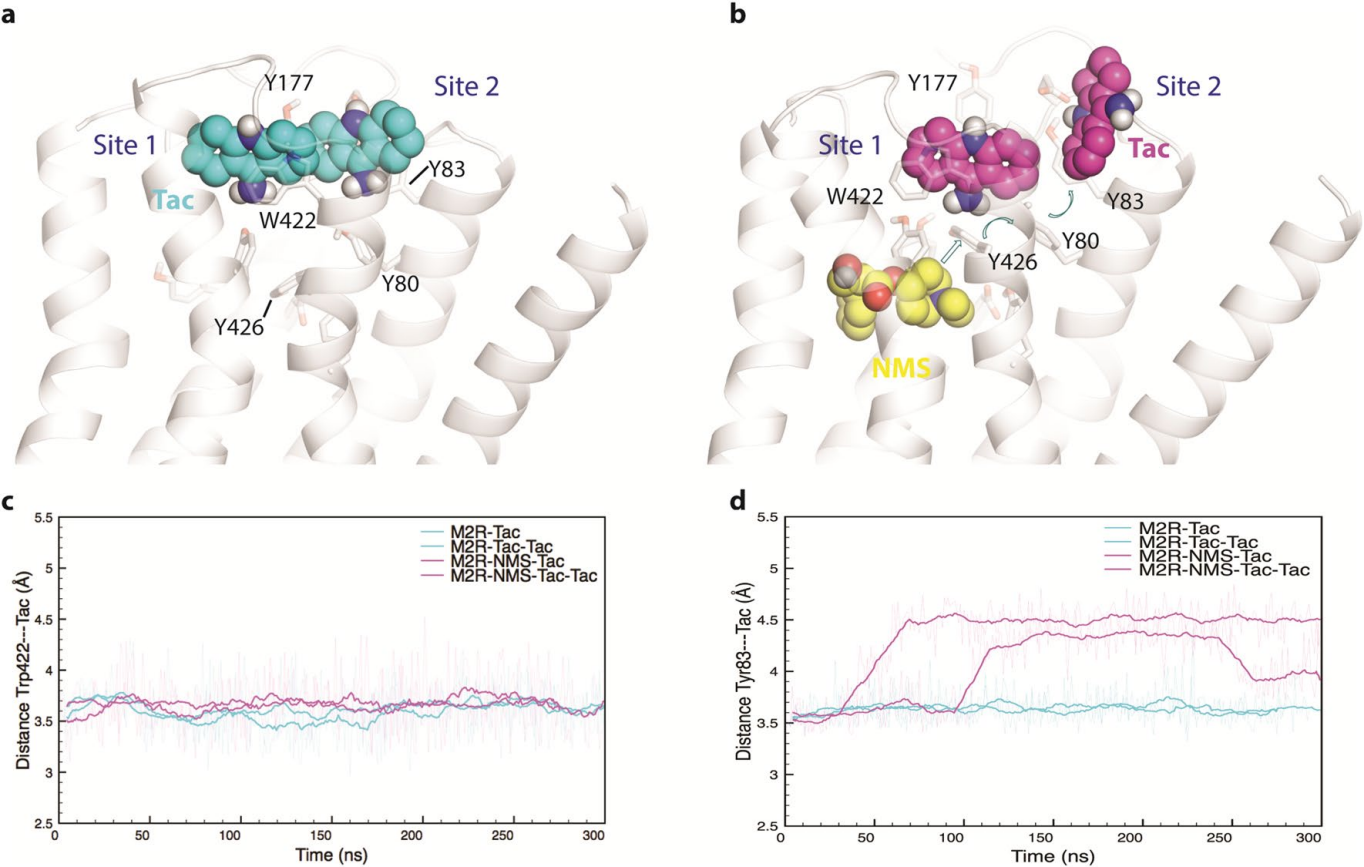
Molecular dynamics of tacrine at the M_2_ receptor. (**a**,**b**) The most frequently observed poses adopted by tacrine at the allosteric site are shown for a monomer of the wild-type receptor in which the orthosteric site is vacant (**a**) or occupied by NMS (**b**). In the otherwise vacant receptor, two molecules of tacrine (cyan) occupy two sites defined by interactions with Tyr^177^ and Trp^422^ (Site 1) and with Tyr^80^ and Tyr^83^ (Site 2). In the receptor occupied by NMS (yellow), the disruption of Site 2 forces a different and unstable orientation of tacrine in that site while having little effect on the binding of tacrine at Site 1 (purple). These computations were performed using PyMOL (The PyMOL Molecular Graphics System, Version 2.0 Schrödinger, LLC; http://www.pymol.org/) (**c**) The stability of tacrine at Site 1 was monitored as the distance from center to center between the pyridine ring of tacrine and the indole ring of Trp^422(7.35)^, which did not change over the production period of 300 ns. (**d**) The stability of tacrine at Site 2 was monitored as the distance from center to center between the pyridine ring of tacrine and the phenyl ring of Tyr^83(2.64)^. Tacrine adopts a stable pose at Site 2 when the orthosteric site is vacant, but the interaction is destabilized in the presence of NMS. Figures (**c**,**d**) were produced in Plot2 version 2.6.11 (https://apps.micw.org/apps/plot2/).

Two sites were identified in the absence of NMS (Fig. 5a). The first allows for stacking interactions with Trp^422(7.35)^ and Tyr^177(EL2)^ (Site 1), and the second allows for interactions with Tyr^80(2.61)^, Tyr^83(2.64)^, and Glu^175(EL2)^ (Site 2). In the crystal structure 4MQT, the same two pairs of aromatic residues (i.e., Trp^422(7.35)^ and Tyr^177(EL2)^, and Tyr^80(2.61)^ and Tyr^83(2.64)^) are seen to make stabilizing contacts with the allosteric modulator LY2119620^20^. Also, mutation of either Trp^422^ or Tyr^177^ is known to preclude the binding of allosteric ligands^21^. Both sites lie in the vestibule of the orthosteric site, near the extracellular surface (Fig. 5a), and in each case the binding of tacrine is stable over the 300 ns period of the simulations (Fig. 5c). The stability at Site 1 appears to derive from π–π/NH–π stacking interactions between the bi-aromatic quinoline core of tacrine and the aromatic rings of Tyr^177^ and Trp^422^. The stability at Site 2 derives from π–π interactions between the cyclopropyl amide chain of tacrine and Tyr^80^ and Tyr^83^. Similar interactions occur with LY2119620^20^, which contains a bi-aromatic thienopyridine core analogous to the bi-aromatic quinoline core of tacrine.

Binding at Site 2 is disrupted upon occupancy of the orthosteric site by NMS, which pushes Tyr^426^ toward the extracellular surface (Fig. 5b). That in turn displaces Tyr^80^, thereby destabilizing the interaction of tacrine with Site 2 (Fig. 5d). Stable binding of tacrine therefore is restricted to Site 1, which is largely unaffected by NMS.

### Identification of allosteric contacts by site-directed mutagenesis

**Figure 6:**
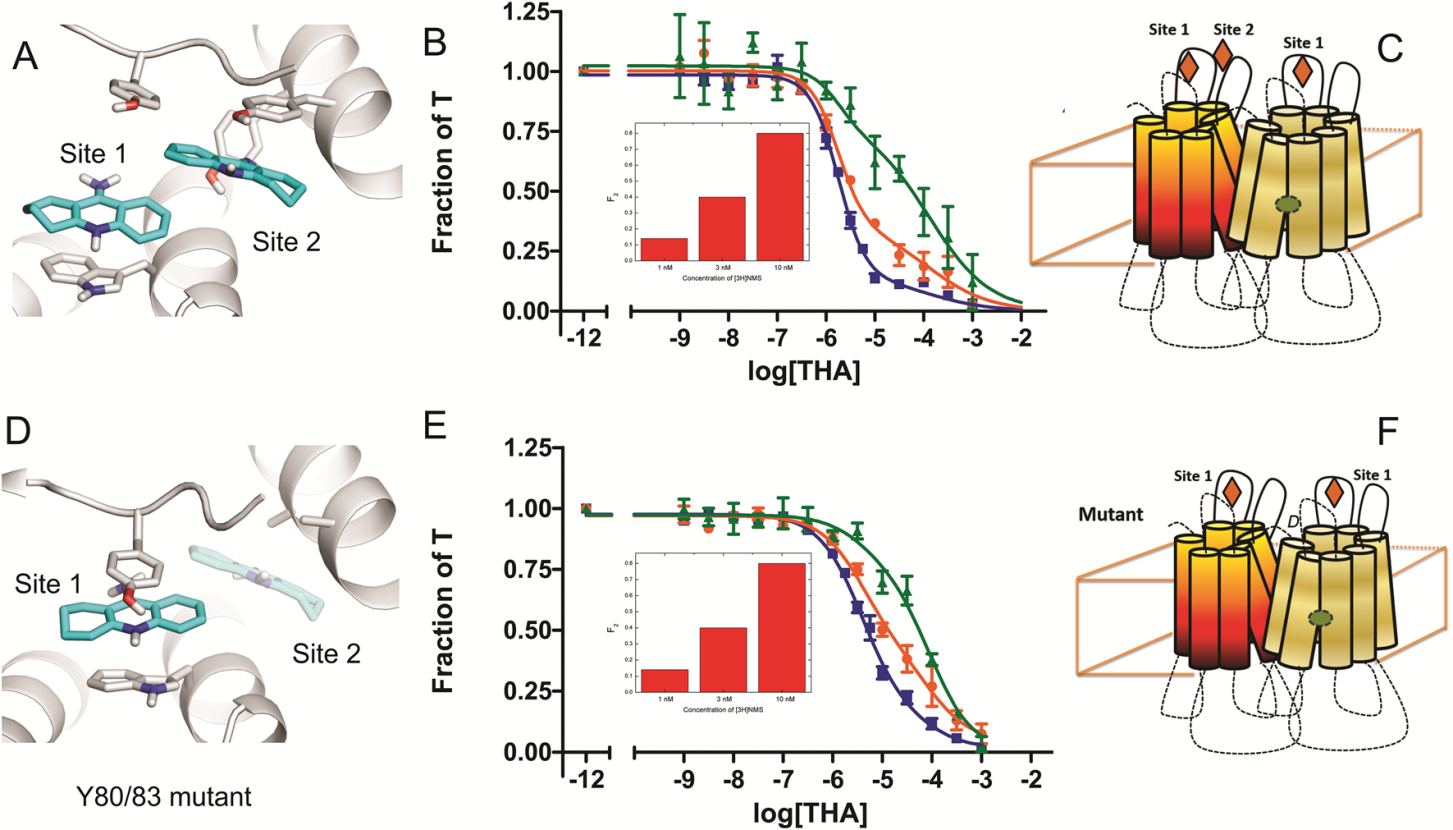
Structural and functional consequences of mutations at the allosteric site of the M_2_ receptor. The wild-type receptor (**A**–**C**) and a mutant in which allosteric Site 2 was eliminated by the substitution of alanine for tyrosine at positions 80 and 83 (**D**–**F**) were compared in molecular dynamics simulations and in binding assays on membranes from CHO cells. (**A**,**D**) Simulations of receptor-bound tacrine as viewed from a position above the extracellular surface. Two allosteric sites are available on an otherwise vacant protomer of the wild-type receptor (Sites 1 and 2) (**A**), whereas only one site is available on the mutant (i.e., Site 1) (**D**) or an NMS-occupied protomer (not shown). These computations were performed using PyMOL (The PyMOL Molecular Graphics System, Version 2.0 Schrödinger, LLC; http://www.pymol.org/). (**B**,**E**) Binding of tacrine. Membranes from CHO cells expressing the wild-type receptor (**B**) or the mutant (**E**) were mixed simultaneously with [^3^H]NMS (filled square, 1 nM; filled circle, 3 nM; filled triangle, 10 nM) and tacrine at the concentrations shown on the abscissa. Binding was measured after equilibration of the mixture for 21 h at 24 °C, and the data were analyzed in terms of Eq. (3) (*n* = 2). The lines in the figure depict the fitted curves, and the values of *F*_2_ are shown in the insets. The fitted parametric values are listed in Table S10. (**C**,**F**) Depictions of the interaction between tacrine (open rhombus) and a dimeric receptor in which the orthosteric site of one protomer is occupied by NMS (open circle). In the wild-type receptor (**C**), the NMS-free protomer can bind two molecules of tacrine (Sites 1 and 2), whereas an NMS-occupied protomer can bind only one (Site 1). In the mutant (**F**), only one molecule of tacrine can bind (Site 1) irrespective of NMS.

Molecular dynamics simulations (Fig. 5) and apparent cooperativity in the inhibitory effect of tacrine (i.e., *n*_H(1)_ > 1) (Figs. 2 and 3, Tables S7–S9) suggest that two allosteric sites are available on an otherwise vacant receptor. To test this prediction, the tyrosine residues associated with allosteric Site 2 were replaced by alanine (i.e., Y80A and Y83A). The wild-type M_2_ receptor and the mutant were expressed separately in CHO cells, and receptor-containing membranes were compared for the binding of tacrine at three concentrations of [^3^H]NMS. The mutations had little effect on the affinity of [^3^H]NMS (Eq. 2, wild-type, *K* = 0.085 ± 0.010 nM, *n*_H_ = 1.02 ± 0.03; mutant, *K* = 0.17 ± 0.02 nM, *n*_H_ = 1.02 ± 0.03). The inhibitory effect of tacrine was biphasic throughout (Fig. 6B,E), and the data were analyzed according to Eq. (3) (*n* = 2). The fitted curves are shown in Fig. 6, and the parametric values are listed in Table S10.

The wild-type receptor in CHO membranes resembled that extracted from *Sf*9 cells (cf, Figs. 3C,E and 6B). An increase in the level of occupancy by [^3^H]NMS was accompanied by an increase in the fraction of sites exhibiting low affinity for tacrine (*F*_2_). There was little or no effect on the affinity or the Hill coefficient at either class of sites. The Hill coefficient significantly exceeds 1 at the sites of high affinity (i.e., *n*_H(1)_ = 1.53) (*P* < 0.01) but not at those of low affinity (*n*_H(2)_ = 1) (*P* > 0.2) (Table S10). The mutant resembled the wild-type receptor in the effect of [^3^H]NMS on *F*_2_, which increased with the concentration of the radioligand with no accompanying change in the affinity of tacrine or the Hill coefficient at either class of sites. The Hill coefficient was 1 at the sites of lower affinity, as in the wild-type receptor; in contrast, the Hill coefficient at the sites of higher affinity decreased from 1.53 in the wild-type receptor to 1 in the mutant (Table S10).

These effects are consistent with the notion that the biphasic inhibitory behavior of tacrine derives from interactions within an oligomer and that the value of *n*_H_ at the sites of higher affinity is indicative of two linked allosteric sites on a constituent protomer with a vacant orthosteric site. Removal of the second allosteric site by the substitution of alanine for tyrosine at positions 80 and 83 was without effect on the oligomeric status of the receptor, as indicated by the retention of biphasic inhibition, but it precluded the positive homotropic cooperativity that otherwise gives rise to a Hill coefficient greater than 1.

## Discussion

The complex effects of small-molecule allosteric modulators indicate that oligomers of the M_2_ muscarinic receptor have functional relevance^7^. Monomers are comparatively limited in their potential for interactions between allosteric and orthosteric ligands. Oligomers allow for cooperativity through linked protomers and therefore offer additional mechanistic pathways for allosteric modulation. Such additional interactions are required to account for the heterotropic allosteric effects of gallamine and strychnine on the binding of [^3^H]NMS and [^3^H]QNB, and the minimum oligomeric size appears to be a tetramer^7,19^. Cooperativity between different ligands at two distinct sites (i.e., allosteric and orthosteric) can be identified with some confidence in the kinetics of dissociation or increased binding at equilibrium; in contrast, cooperativity between successive equivalents of the same ligand is less readily discerned owing to data that tend to be ambiguous. Such ambiguity is avoided or at least reduced with atypical allosteric modulators such as tacrine, where Hill coefficients greater than 1 argue for positive cooperativity between successive equivalents of the ligand^13,14^.

In this study, we have sought to understand the molecular mechanism of action of tacrine and how it relates to the oligomeric state of the receptor. Our approach has involved molecular dynamics based on a monomer and binding studies in various preparations of monomers and oligomers: namely, monomers purified from *Sf*9 cells^17^, oligomers in solubilized extracts from *Sf*9 cells and porcine atria^22^, and known or presumed oligomers in membranes from *Sf*9 cells, CHO cells, and porcine atria^23^. The results from atrial preparations show the properties of the receptor in a natural tissue and speak to their biological relevance.

In common with previous studies with gallamine and strychnine^7^, various effects of tacrine in experiments conducted at equilibrium point to interactions within and between the protomers of an oligomer. Neither the tacrine-induced decrease in the apparent capacity for [^3^H]NMS (Fig. 1A) or [^3^H]QNB (Fig. 1B) nor the inhibitory effect of tacrine on the binding of [^3^H]NMS (Figs. 2, 6B) can be explained in terms of single allosteric and orthosteric sites. Data on the binding of either radioligand require at least two orthosteric sites, and the inhibitory effects of tacrine point to least three allosteric sites (Tables S7, S10). At least two of the latter can be inferred from the biphasic nature of the inhibition; in addition, Hill coefficients consistently greater than 1 suggest that the high-affinity component itself results from positive homotropic cooperativity in the binding of tacrine to at least two allosteric sites. Evidence for positivity cooperativity also can be seen in the effect of tacrine on the rate of dissociation of [^3^H]NMS, which similarly exhibits Hill coefficients greater than 1 as shown here (Fig. S8) and previously^13,14^.

The effects of tacrine do not appear to derive from experimental artifacts. Small organic molecules are known to aggregate in some cases^24^, but that seems unlikely to have been a factor here. Tacrine is positively charged at physiological pH (p*K*_a_ = 9.95; PubChem CID 1935^25^); also, its atypical effects on the time-dependent and equilibrium binding of each orthosteric ligand were retained upon solubilization of the receptor in digitonin. Both the positive charge and the detergent are expected to discourage aggregation of the ligand, although aggregation and other alternatives to the notion of interactions within oligomers cannot be ruled out.

Another concern relates to the attainment of equilibrium, which is essential to the interpretation of the data in some experiments and was ensured by lengthy periods of incubation. Prolonged incubation was associated with some inactivation of the receptor at higher temperatures, but there was no consistent or substantive change in the parameters of interest (Table S7). Also, the biphasic inhibitory effect that developed upon the addition of tacrine and [^3^H]NMS to M_2_ receptor in atrial extracts appeared to be the same regardless of the order of mixing, as expected for a system approaching equilibrium.

The selectivity of tacrine is unknown at the highest concentrations used in some experiments (i.e., 3–10 mM). Orthosteric ligands can bind to the allosteric site^26^, albeit weakly. It is conceivable that an allosteric ligand might bind weakly to the orthosteric site, but that seems unlikely to account for the observed atypical effects. The decrease in the capacity for [^3^H]NMS and [^3^H]QNB brought about by tacrine at a concentration of 3.2 mM cannot be attributed to competition for the orthosteric site (Fig. 1, Equation S9). Also, the dissociation of [^3^H]NMS was slowed progressively by tacrine at concentrations up to 1 mM, and the inhibitory potency of tacrine did not increase with increasing concentrations of the orthosteric ligand. These considerations suggest that the effects of tacrine are wholly non-competitive and therefore allosteric.

Essentially the same effects were observed with receptor in native membranes from porcine atria and heterologous cell expression systems and in corresponding solubilized preparations extracted from *Sf*9 cells, CHO cells and porcine atria. The similarities indicate that the properties of the solubilized receptor were present in the membrane and not introduced as a consequence of solubilization. Also, the level of expression of M_2_ receptor in sarcolemmal membranes from porcine atria approaches that in membranes from *Sf*9 cells^27^.

The kinetics of the interaction between the receptor and each radioligand support conclusions based on the rate of dissociation of [^3^H]NMS and studies at equilibrium. Such effects are more pronounced with the solubilized receptor^19^ than with the membrane-bound receptor^28^. The complexity tends to emerge more rapidly with the receptor in solution. In solubilized extracts, neither [^3^H]QNB nor [^3^H]NMS associates with the receptor in a manner that can be described by a single rate constant (Figs. 1D, S3A). Mechanistic analyses based on a model of a dimeric receptor (Scheme 1) provide at least a first approximation of the data in the case of [^3^H]QNB (Fig. 1D), but the model fails to describe the increase and subsequent decrease in the binding of [^3^H]NMS (Fig. S3A).

The kinetics of binding are complex, the most striking feature being an overshoot that is especially prominent with [^3^H]NMS and less so with [^3^H]QNB. In each case, the time-course of binding appears to result from reversible conformational changes within the receptor. Constraints imposed by the mono-exponential kinetics of dissociation (Fig. S6) limit the ability of a dimer to account for the kinetics of association, as illustrated by simulations in which those constraints are lifted to allow for multiphasic dissociation (Fig. S3, Table S4). Also, the binding assays included a negative allosteric modulator and an inverse agonist, at least in the case of [^3^H]NMS. The receptor therefore was driven to the inactive state, which is consistent with the assumption of a single state in our analyses. Anomalous kinetics also can arise if a ligand added during expression of the receptor is not removed prior to the assays^29^, but no ligand was present during expression of the M_2_ receptor. In addition to slowing the association and dissociation of [^3^H]NMS and [^3^H]QNB, tacrine simplified the time-course of association in each case. Both traces were reduced to a single exponential, a change that is especially pronounced in the case of [^3^H]NMS (cf. Figs. S3A and S6). This action of tacrine further supports the notion that time-dependent changes in the binding of the radioligand result from shifts in the distribution of receptors among different liganded states and that the process is subject to allosteric modulation.

At equilibrium, tacrine identifies sites of high and low affinity when measured at different concentrations of [^3^H]NMS and [^3^H]QNB; the inhibitory effect of tacrine was biphasic throughout (Fig. 3). Whereas the inhibitory potencies were largely unaffected, the fraction of sites apparently of low affinity tracked the level of occupancy by the radioligand in the absence of tacrine (Table S9). This pattern suggests that the sites of higher affinity for tacrine are located on protomers with vacant orthosteric sites and that those of weaker affinity are located on protomers with occupied orthosteric sites. Inhibition via the sites of higher affinity therefore appears to be intermolecular, and that via the sites of weaker affinity is intramolecular. Essentially the same pattern has been observed previously with gallamine^19^.

Purified monomers of the M_2_ receptor were used to distinguish between properties that are intrinsic to a dissociated protomer and those that emerge as a result of oligomerization. In that preparation, the dose-dependent effects of tacrine on the rate of dissociation of [^3^H]NMS and on the binding of [^3^H]NMS at equilibrium gave Hill coefficients indistinguishable from 1 (Fig. 4). Monomers therefore behaved as expected for an interaction between single allosteric and orthosteric sites, in marked contrast to the complex behavior observed in preparations of oligomers. The biphasic inhibitory behavior was lost, as were Hill coefficients greater than 1 and the implied positive cooperativity. The value of 1 obtained for *n*_H_ in the effect of tacrine of the dissociation of [^3^H]NMS suggests that a receptor with an occupied orthosteric site binds only one equivalent of the allosteric ligand. That concurs with the suggestion that inhibition at the sites of lower affinity, which generally had Hill coefficients near 1, is an intramolecular effect whereas that at the sites of higher affinity, where the Hill coefficient exceeded 1, is an intermolecular effect.

The two putative sites for tacrine on an otherwise vacant protomer were identified by means of molecular dynamics simulations. The first involves interactions with Tyr^177^ and Trp^422^ (Site 1), and the second involves interactions with Tyr^80^ and Tyr^83^ (Fig. 5). The site most disrupted by an orthosteric ligand is Site 2 (*i.*e., Y80 and Y83), which therefore is likely to engage exclusively in intermolecular allosteric interactions. Site 2 therefore was eliminated by the replacement of Tyr^80^ and Tyr^83^ (*i.e.*, Y80A and Y83A), and the mutant was expressed in CHO cells and examined for the effect of tacrine on the binding of [^3^H]NMS (Fig. 6, Table S10). As with the wild-type receptor, the inhibitory profile was biphasic at each of three different concentrations of [^3^H]NMS; also, the fraction of sites of lower affinity for tacrine increased with the level of occupancy by the radioligand. Properties of the purified monomer indicate that such effects are characteristic of an oligomer. In contrast to the wild-type receptor, however, the Hill coefficient for tacrine at the sites of higher affinity in the mutant was 1. The decrease in the value of *n*_H_ indicates that the mutations reduced the stoichiometry of binding from 2 to 1. The mutations thereby preclude positive cooperativity at the sites of higher affinity for tacrine, but they are without effect on other interactions within the oligomer or on its oligomeric status.

The four tyrosine residues identified by molecular dynamics are located near the extracellular surface in a shallow site that can accommodate two independent molecules of tacrine, one molecule of a fused bi-molecule of tacrine or one molecule of gallamine. The latter is a prototypical allosteric ligand that has been shown to interact with residues in Sites 1 and 2 through its three cationic ammonium groups. Two molecules of tacrine bound to Sites 1 and 2 in the M_2_ muscarinic receptor are analogous to BQZ12 bound to the M_1_ muscarinic receptor^30^.

GPCR-mediated signalling and its modulation by allosteric ligands often is seen in terms of monomers. Although multimeric forms of many GPCRs have been identified, their functional relevance has yet to be established^18^. Among GPCRs of the rhodopsin-like family, that uncertainty exists because monomers display at least some of the functionality of receptors in native tissues (e.g.,^31^). We previously have shown that the M_2_ muscarinic receptor forms tetramers in CHO cells^27^and can be purified from *Sf*9 and CHO cells as a heteromeric complex of four receptors and four holo-G_i1_ proteins^6^. Tetramers of the M_2_ receptor are required for the full expression of characteristic binding properties linked to efficacy^17,26^, and muscarinic agonists have been shown to promote the coupling of oligomers of the M_2_ receptor with oligomers of G_i1_ in CHO cells^32^. Such observations suggest that oligomers are integral to the process of signalling.

The present results indicate that the recognized functionality of oligomers can be expanded to include modulation by atypical allosteric ligands. The characteristic effects of tacrine on the binding of orthosteric antagonists to the M_2_ receptor were observed with oligomers but not monomers. The nature of those effects indicate that the receptor was an oligomer larger than a dimer, and observations such as those recounted above suggest that it most likely was a tetramer. Tacrine seems to take full advantage of the mechanistic network that is created by the potential for cooperative interactions within such a complex: namely, homo- and heterotropic cooperativity with respect to the ligands, and intra- and intermolecular cooperativity with respect to the constituent protomers. At the level of a protomer with a vacant orthosteric site, two molecules of tacrine bind to the allosteric site with high affinity and positive cooperativity (i.e., *n*_H_ > 1); occupancy of the orthosteric site reduces the affinity of tacrine for the allosteric site as well as the stoichiometry of binding, from two molecules of tacrine to one. All such effects are intramolecular. At the level of an oligomer, intermolecular interactions permit tacrine to bind with higher affinity and homotropic cooperativity to the allosteric site of an otherwise vacant protomer while inhibiting the binding of an antagonist to the orthosteric site of another, linked protomer. The growing realization that many GPCRs contain allosteric sites^33^, the potential for allosteric modulators in therapeutic intervention^34^ and the ubiquity of oligomers suggest that the properties described here have broad implications.

## Methods

Experimental procedures. Details regarding chemicals and other materials, the production and purification of M_2_ receptor from CHO and *Sf*9 cells, and assays for the binding of radioligands are described in the Supplementary Information (Section S3). Details regarding the procedures for molecular dynamics are described below and in Section S3.

### Analysis of binding data

Analyses of binding data from kinetic and equilibrium experiments were performed as described previously^19^. Briefly, the association of [^3^H]QNB or [^3^H]NMS with the receptor over time was analyzed empirically as a sum of exponentials according to Eq. (1a), in which *B*_obsd_ is the total binding of the radioligand at time t, and *B*_t=0_ and *B*_t→∞_ represent the initial and asymptotic levels of binding, respectively; *k*_obsd(*j*)_ is the rate constant for binding to those sites that constitute the fraction *F*_*j*_ of all labeled sites.

1a $$B_{{{\text{obsd}}}} = \left( {B_{{{\text{t}} = {0}}} - B_{{{\text{t}} \to \infty }} } \right) \cdot \sum\limits_{j = 1}^{n} {F_{j} \left( {1 - e^{{ - k_{{{\text{obsd}}(j)}} {\text{t}}}} } \right)} + B_{{{\text{t}} \to \infty }}$$

The net dissociation of [^3^H]QNB or [^3^H]NMS over time conformed throughout to a single exponential and was analyzed according to Eq. (1b), in which the parameters and variables are as described above.

1b $$B_{{{\text{obsd}}}} = \left( {B_{{{\text{t}} = 0}} - B_{{{\text{t}} \to \infty }} } \right) \cdot e^{{ - k_{{{\text{obsd}}}} {\text{t}}}} + B_{{{\text{t}} \to \infty }}$$

Time-courses of dissociation at one or more concentrations of the allosteric ligand were accompanied in the same experiment by a control without the allosteric ligand. The data from all traces were analyzed in concert with a single value of *B*_t→∞_ and separate values of *k*_obsd_ and *B*_t=0_, which was without appreciable effect on the sum of squares (*P* > 0.05). The value of *k*_obsd_ in the absence of the allosteric ligand was designated *k*_0_ and used to normalize the value of *k*_obsd_ in the presence of the allosteric ligand to obtain the ratio *k*_obsd_/*k*_0_.

Data on the association and dissociation of [^3^H]QNB and [^3^H]NMS also were subjected to mechanistic analyses in terms of Scheme 1 (Fig. 1), in which the radioligand binds to a dimer of receptors. Further details are described below and in the Supplementary Information (SI, Section S1).

Binding at graded concentrations of *N*-[^3^H]methylscopolamine or [^3^H]quinuclidinylbenzilate was analyzed in terms of Eq. (2), in which *B*_max_ represents maximal specific binding of the radioligand (P); *B*_obsd_ and *B*_sp_ represent total and specific binding, respectively, at the total concentration [P]_t_. The parameter *K* is the concentration of unbound radioligand that corresponds to half-maximal specific binding, and *n*_H_ is the Hill coefficient; *NS* is the fraction of unbound radioligand that appears as nonspecific binding. Equation (2) was solved numerically as described previously^35^.

2 $$B_{{{\text{obsd}}}} = B_{{{\text{max}}}} \sum\limits_{j = 1}^{n} {\left( {\frac{{F_{j} \left( {[{\text{P]}}_{{\text{t}}} - B_{{{\text{sp}}}}{^{{n_{{\text{H}}} }} }} \right)}}{{K^{{n_{{\text{H}}} }} + \left( {[{\text{P]}}_{{\text{t}}} - B_{{{\text{sp}}}} )^{{n_{{\text{H}}} }} } \right)}}} \right)} + NS\left( {[{\text{P]}}_{{\text{t}}} - B_{{{\text{sp}}}} } \right)$$

Dose-dependent effects of tacrine (T) on the rate of dissociation of [^3^H]NMS or [^3^H]QNB (*k*_obsd_/*k*_0_), or on the level of total binding at a specified time (*B*_obsd_), were analyzed empirically in terms of Eq. (3).

3 $$Y_{{{\text{obsd}}}} = Y_{{[{\text{T]}} \to \infty }} + (Y_{{[{\text{T]}} = {0}}} - Y_{{[{\text{T]}} \to \infty }} )\sum\limits_{j = 1}^{n} {\frac{{F_{j} K_{j}^{{n_{{{\text{H(}}j)}} }} }}{{[{\text{T]}}_{{\text{t}}}^{{n_{{{\text{H(}}j)}} }} + K_{j}^{{n_{{{\text{H(}}j)}} }} }}}$$

The variable *Y*_obsd_ represents the value of *k*_obsd_/*k*_0_ or *B*_obsd_ at the total concentration [T]_t_ of tacrine, and the parameters *Y*_[T]=0_ and *Y*_[T]→∞_ represent the value of *Y*_obsd_ in the absence of the allosteric ligand and at saturating concentrations, respectively. The allosteric-sensitive component of *Y* is described as a sum of *n* Hill terms; the difference *Y*_[T]=0_ – *Y*_[T]→∞_ is the net change effected by T, *F*_*j*_ is the fractional contribution of term *j* (*i.e.*, $\sum\nolimits_{j = 1}^{n} {F_{j} } = 1$), *n*_H(*j*)_ is the corresponding Hill coefficient, and *K*_*j*_ is the total concentration of T that yields a half-maximal signal at fraction *j*. The total and free concentrations of tacrine were essentially the same under the conditions of the assays.

### Statistical procedures

All equations were fitted to the data by nonlinear regression^36^. Equilibrium constants and potencies were optimized throughout on a logarithmic scale, and rate constants were optimized on a linear scale. Multiple sets of data from replicated experiments generally were analyzed in concert. Parameters that were expected to be the same from one experiment to another were optimized as single values common to all of the data in the analysis (e.g., *K*_*j*_, *n*_H_, and *F*_*j*_ in Eqs. (2) and (3); *K*_*j*_, *k*_±*j*_, and *α* in Scheme 1). Other parameters were assigned separately to the data from each experiment (*e.g.*, *B*_max_, *NS*, *Y*_[T]=0_, and *Y*_[T]→∞_ in Eqs. (2) and (3); [R]_t_ in Scheme 1).

The effects of various constraints on the weighted sum of squares were assessed by the means of the *F*-statistic. Weighting of the data and other statistical procedures were performed as described previously^35^. Mean parametric values calculated from independent estimates are presented together with the standard error.

### Scaling and presentation of data

Results of analyses involving multiple sets of data from replicated experiments are shown with reference to a single fitted curve. To obtain the values plotted on the *y*-axis, estimates of observed binding (*Y*_obsd_) or specific binding (*Y*_sp_) were adjusted according to the equation $Y^{\prime} = Y\{ f(\overline{{\mathbf{x}}}_{i} ,\overline{{\mathbf{a}}} )/f({\mathbf{x}}_{i} ,{\mathbf{a}})\}$^37^. The function *f* represents the fitted model. The vectors **x**_*i*_ and **a** represent the independent variables at point *i* and the fitted parameters for the set of data under consideration; $\overline{{\mathbf{x}}}_{i}$ and $\overline{{\mathbf{a}}}$ are the corresponding vectors in which values from various experiments have been replaced by the means for all experiments included in the analysis.

### Mechanistic models

Binding of a ligand (L) to a potentially asymmetric and cooperative dimer of receptors (${\text{R}}_{{}}^{1} {\text{R}}_{{}}^{2}$) occurs in a stepwise manner, as depicted in Scheme 1. The kinetically determined description of the model comprises four differential equations, one for each species of receptor (i.e., ${\text{R}}_{{}}^{1} {\text{R}}_{{}}^{2} ,$ ${\text{R}}_{{\text{L}}}^{{1}} {\text{R}}_{{}}^{2} ,$ ${\text{R}}_{{}}^{{1}} {\text{R}}_{{\text{L}}}^{2} ,$ and ${\text{R}}_{{\text{L}}}^{{1}} {\text{R}}_{{\text{L}}}^{2}$). The values of the rate constants were estimated by fitting the system of differential equations to data on the binding of [^3^H]QNB or [^3^H]NMS over time. The integrals were computed numerically using the ODS23 subroutine in Matlab 2012, and total specific binding (*B*_sp_) was taken as the sum of all receptor-bound ligand (i.e., $B_{{{\text{sp}}}} {\text{ = [R}}_{{\text{L}}}^{{1}} {\text{R}}_{{}}^{2} {]}$ + ${\text{[R}}_{{}}^{{1}} {\text{R}}_{{\text{L}}}^{2} {]}$ + ${\text{2[R}}_{{\text{L}}}^{{1}} {\text{R}}_{{\text{L}}}^{2} ]$). Parametric values were optimized according to the Marquardt–Levenberg algorithm. Further details are described in the Supplementary Information (Section S1).

**Scheme 2:**
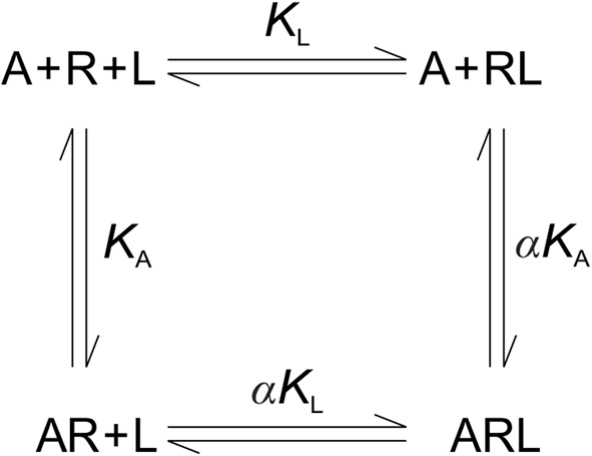
Heterotropic Cooperativity in the Binding of Orthosteric and Allosteric Ligands to a Monomeric Receptor in a System at Equilibrium.

An orthosteric ligand (L) and an allosteric ligand (A) bind to topographically distinct sites on a monomeric receptor as depicted in Scheme 2, where *K*_L_ and *K*_A_ are the equilibrium dissociation constants of L and A for the vacant receptor. Either ligand may bind to a receptor occupied by the other to form the ternary complex ARL. The cooperativity factor *α* gives the effect of Ligand A on the affinity of Ligand L and vice versa. Further details are described in the Supplementary Information (Section S1).

### Molecular dynamics simulations

#### System setup

Simulations of the muscarinic M_2_ receptor (M2R) were based on a crystal structure of the receptor in complex with QNB (3UON.pdb). QNB was either removed or replaced with NMS, and one or two tacrine molecules of tacrine were placed in the allosteric site at positions designated Site 1 and Site 2. Seven conditions were simulated: NMS-bound M2R with no allosteric ligand and in complex with tacrine at Site 1, Site 2, or both; and M2R with no orthosteric ligand and tacrine at Site 1, Site2, or both. The placement of tacrine was guided by the crystal structure of active-state M2R in complex with iperoxo and the allosteric modulator LY2119620 (4MQT.pdb). Hydrogen atoms were added using Prime (Schrödinger, Inc.), and the *N*- and *C*-termini were capped with neutral groups (i.e., acetyl and methylamide, respectively). Titratable residues were left in their dominant protonation state at pH 7.0. All aspartate residues were deprotonated, as is expected in the inactive state of GPCRs, and the tertiary amines of the ligands were protonated. The prepared protein structures were aligned on their transmembrane helices to the Orientation of Proteins in Membranes (OPM)^15^ structure of 3UON.pdb, and internal waters were added with Dowser^6^. The structures then were inserted into a pre-equilibrated palmitoyl-oleoyl-phosphatidylcholine (POPC) bilayer, solvated with 0.15 M NaCl in explicitly represented water, and neutralized by removing sodium ions.

#### Force-field parameters

We used the CHARMM36 parameter set for protein molecules, lipid molecules, and salt ions, and the CHARMM TIP3P model for water; protein parameters incorporated CMAP terms. Parameters for ligands were generated using the CHARMM General Force Field (CGenFF)^38^ with the ParamChem server (paramchem.org, Version 1.0.0). Hydrogen mass repartitioning was employed to enable 4 fs.

#### Simulation protocol

Simulations were performed on graphics processing units using the CUDA version of PMEMD (Particle Mesh Ewald Molecular Dynamics) in Amber15. Prepared systems were minimized and then equilibrated as follows. The system was heated using the Langevin thermostat from 0 to 100 K in the NVT ensemble over 12.5 ps with harmonic restraints of 10.0 kcal mol^−1^ Å^−2^ on the non-hydrogen atoms of lipid, protein and ligand, and with initial velocities sampled from the Boltzmann distribution. The system then was heated to 310 K over 125 ps in the NPT ensemble with semi-isotropic pressure coupling and a pressure of one bar. Further equilibration was performed at 310 K with harmonic restraints on the protein and ligand starting at 5.0 kcal mol^−1^∙Å^−2^ and reduced by 1.0 kcal mol^−1^ Å^−2^ in a stepwise fashion every 2 ns, for a total of 10 ns of additional restrained equilibration.

Simulations were conducted in the NPT ensemble at 310 K and 1 bar, using a Langevin thermostat and Monte Carlo barostat. In each simulation, we performed 5 ns of unrestrained equilibration followed by a production run of at least 280 ns. Simulations used periodic boundary conditions and a time-step of 4.0 fs. Bond lengths to hydrogen atoms were constrained using SHAKE. Non-bonded interactions were cut off at 9.0 Å, and long-range electrostatic interactions were computed using the particle mesh Ewald (PME) method with an Ewald coefficient (*β*) of approximately 0.31 Å and B-spline interpolation of order 4. The FFT grid size was chosen such that the width of a grid cell was approximately 1 Å.

#### Simulation analysis

Trajectory snapshots were saved every 100 ps during production simulations. Trajectory analysis was performed using VMD (Visual Molecular Dynamics) and CPPTRAJ, and visualization was performed using VMD. Two metrics were used to determine the stability of tacrine at its two sites: at Site 1, the distance between the center of the pyridine ring of tacrine and that of the indole ring of Trp-422^7.35^; at Site 2, the distance between the center of the pyridine ring of tacrine and that of the phenol ring of Tyr-80^2.61^. Figures were rendered using PyMol.

## Supplementary Information

Supplementary Information.

## Abbreviations

GPCR: G protein-coupled receptor
HEPES: 4-(2-Hydroxyethyl)-1-piperazineethanesulfonic acid
PBS: Phosphate-buffered saline
PMSF: Phenylmethanesulfonyl fluoride
NMS: *N*-methylscopolamine
QNB: Quinuclidinylbenzilate
