# Supplementary material for: Allostery of atypical modulators at oligomeric G protein-coupled receptors
Source: Sci Rep. 2021 Apr 29;11:9265. doi: 10.1038/s41598-021-88399-x (PMC8085029; doi:10.1038/s41598-021-88399-x)
Supplement: Supplementary file 1 — Supplementary Information. [file 41598_2021_88399_MOESM1_ESM.docx]

**Supplementary Information**

Allostery of Atypical Modulators at Oligomeric G Protein-coupled Receptors

Rabindra V. Shivnaraine^1,2*^, Brendan Kelly^5^, Gwendolynne Elmslie^3^, Xi-Ping Huang^3,4^, Yue John Dong^1^, Margaret Seidenberg^3^, James W. Wells^1,*^ and John Ellis^3,*^

^1^Department of Pharmaceutical Sciences, Leslie Dan Faculty of Pharmacy, University of Toronto, Toronto, Ontario M5S 3M2. ^2^Current address: Department of Molecular and Cellular Physiology, Stanford University School of Medicine, Stanford, CA 94305 ^3^Departments of Psychiatry and Pharmacology, Hershey Medical Center, Penn State University College of Medicine, Hershey, PA 17033.  ^4^Current address: Department of Pharmacology, The National Institute of Mental Health Psychoactive Drug Screening Program (NIMH PDSP), Department of Pharmacology, University of North Carolina at Chapel Hill, Chapel Hill, NC 27599. ^5^Departments of Computer Science, Molecular and Cellular Physiology, and Structural Biology, and Institute for Computational and Mathematical Engineering, Stanford University, Stanford, CA 94305, USA

**Section S1: Mechanistic models of asymmetry, homotropic cooperativity, and heterotropic cooperativity**

G protein-coupled receptors (GPCRs) of Family 1 bind agonists and antagonists at a single orthosteric site located within the cluster of seven transmembrane helices^1^. Many GPCRs also form homo-oligomers where packing of the constituent protomers is likely to introduce structural differences between receptors at different locations within the cluster. Such an asymmetry may lead in turn to heterogeneity within an otherwise homogeneous population of orthosteric sites. In the case of a dimer, for example, a ligand might bind to one receptor with higher affinity and to the other with lower affinity (Figure S1). Also, conformational linkages between contiguous protomers might allow for cooperative interactions that contribute to a pre-existing asymmetry or introduce asymmetry into an otherwise symmetric system.

**Figure S1**. An asymmetric dimer in which the constituent protomers differ in their affinity for an orthosteric ligand and engage in homotropic cooperativity between the orthosteric sites.

**Kinetically defined model of asymmetry and homotropic cooperativity within a dimer.** Binding of a ligand (L) to a potentially asymmetric and cooperative dimer of receptors ($\text{R}^{1}\text{R}^{2}$) is depicted in Scheme S1, which is reproduced from Scheme 1 in the parent text. Binding occurs in a stepwise manner, leading first to a monoliganded species (*i.e.*, $\text{R}_{\text{L}}^{1}\text{R}^{2}$or $\text{R}^{1}\text{R}_{\text{L}}^{2}$) and then to the fully occupied dimer ($\text{R}_{\text{L}}^{1}\text{R}_{\text{L}}^{2}$).

Scheme S1


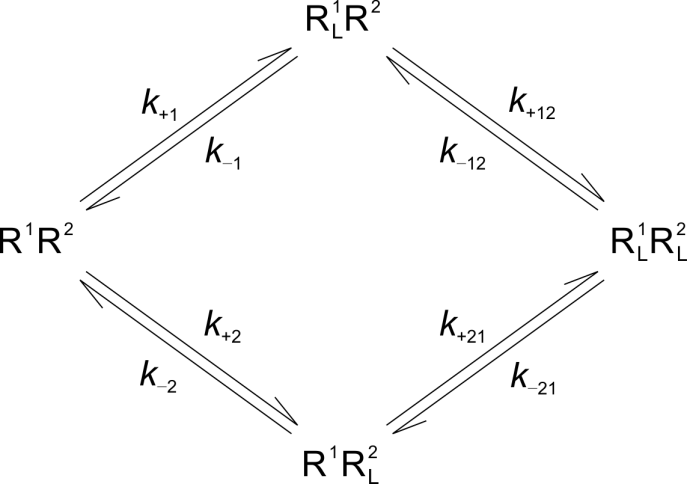


The parameters *k*_−1_ and *k*_+1_ in Scheme S1 are the first- and second-order rate constants for the binding of L to Protomer 1 of the dimer (*i.e.*,R^1^); similarly, *k*_−2_ and *k*_+2_ are the first- and second-order rate constants for the binding of L to Protomer 2 (*i.e.*, R^2^). The parameters *k*_−12_ and *k*_+12_ are the corresponding rate constants for the binding of the second equivalent of L to $\text{R}_{\text{L}}^{1}\text{R}^{2}$, and *k*_−21_ and *k*_+21_ are those for the binding of L to $\text{R}^{1}\text{R}_{\text{L}}^{2}$. The rate constants and corresponding equilibrium dissociation constants (*K*) are related according to Equations S1–S4, where the cooperativity factor *α* ensures that all values are consistent with the principle of microscopic reversibility.

$K_{1}=\frac{\text{[R}^{1}\text{R}^{2}\text{][L]}}{\text{[R}_{\text{L}}^{1}\text{R}^{2}\text{]}}\equiv\frac{k_{-1}}{k_{+1}}$ (S1)

$K_{2}=\frac{\text{[R}^{1}\text{R}^{2}\text{][L]}}{\text{[R}^{1}\text{R}_{\text{L}}^{2}\text{]}}\equiv\frac{k_{-2}}{k_{+2}}$ (S2)

$K_{12}=\frac{\text{[R}_{\text{L}}^{1}\text{R}^{2}\text{][L]}}{\text{[R}_{\text{L}}^{1}\text{R}_{\text{L}}^{2}\text{]}}\equiv\frac{k_{-12}}{k_{+12}}\equiv\alpha K_{2}$ (S3)

$K_{21}=\frac{\text{[R}^{1}\text{R}_{\text{L}}^{2}\text{][L]}}{\text{[R}_{\text{L}}^{1}\text{R}_{\text{L}}^{2}\text{]}}\equiv\frac{k_{-21}}{k_{+21}}\equiv\alpha K_{1}$ (S4)

The value of *α *determines the equilibrium dissociation constant of the second equivalent of L in the presence of the first (*i.e.*, *αK*_1_ or *αK*_2_). In a system at thermodynamic equilibrium, the dimer may be either symmetric (*K*_1_ = *K*_2_) or asymmetric; asymmetry may be intrinsic to the dimer (*K*_1_ ≠ *K*_2_), induced through cooperative effects in the binding of the ligand (*α* ≠ 1), or both. In a kinetically determined system, *α* may affect either or both of the constituent rate constants according to Equations S5 and S6, in which the balance is established by the values of *m* or *n*. Further details on the partitioning of *α* between the rate constants have been described previously^2^.

$\frac{k_{\text{-12}}}{k_{+12}}=\alpha\frac{k_{\text{-1}}}{k_{+1}}\equiv\frac{\alpha^{m+1}k_{\text{-1}}}{\alpha^{m}k_{+1}}\equiv\frac{\left(\frac{1}{\alpha}\right)^{m}k_{\text{-1}}}{\left(\frac{1}{\alpha}\right)^{m+1}k_{+1}}$ (S5) $\frac{k_{\text{-21}}}{k_{+21}}=\alpha\frac{k_{\text{-2}}}{k_{+2}}\equiv\frac{\alpha^{n+1}k_{\text{-2}}}{\alpha^{n}k_{+2}}\equiv\frac{\left(\frac{1}{\alpha}\right)^{n}k_{\text{-2}}}{\left(\frac{1}{\alpha}\right)^{n+1}k_{+2}}$ (S6)

In the present investigation, estimates of the rate constants were obtained by fitting a system of differential equations to data on the time-dependent binding of a radiolabeled ligand. The values were optimized by non-linear regression according to a Levenberg-Marquardt procedure. Rates of association and dissociation were measured with the receptor either vacant or pre-labeled with the radioligand at initial time, and data from both types of experiment were analyzed in concert. Further details regarding the experimental procedures have been described previously^2^.

The model comprises four differential equations, one for each species of receptor (*i.e.*, $\text{R}^{1}\text{R}^{2},$ $\text{R}_{\text{L}}^{1}\text{R}^{2},$ $\text{R}^{1}\text{R}_{\text{L}}^{2},$ and $\text{R}_{\text{L}}^{1}\text{R}_{\text{L}}^{2}$). There was no differential equation for the ligand, which was assumed not to be depleted appreciably through binding to the receptor under the conditions of the experiments described here. The total concentration of receptor was defined as the concentration of protomers [*i.e.*, $\text{[R]}_{\text{t}}\text{ = 2(R}^{1}\text{R}^{2}$+ $\text{R}_{\text{L}}^{1}\text{R}^{2}$+ $\text{R}^{1}\text{R}_{\text{L}}^{2}$ + $\text{R}_{\text{L}}^{1}\text{R}_{\text{L}}^{2})$]. The concentration of each species of receptor at each time was computed by numerical integration in Matlab 2012, and the integrals were calculated using the ODE23s subroutine. Total specific binding (*B*_sp_) was taken as the sum of all receptor-bound ligand; that is, $B_{\text{sp}}\text{= [R}_{\text{L}}^{1}\text{R}^{2}\text{]}$ + $\text{[R}^{1}\text{R}_{\text{L}}^{2}\text{]}$ + $\text{2[R}_{\text{L}}^{1}\text{R}_{\text{L}}^{2}]$.

**Equilibrium and kinetically defined models of cooperativity between one orthosteric site and one allosteric site.** An orthosteric ligand (L) and an allosteric ligand (A) bind to topographically distinct sites on a receptor (R), as depicted in Scheme S2 (**A** and **B**). A promiscuous allosteric ligand also may bind to the orthosteric site, as depicted in Scheme S2**C**. Binding of an orthosteric ligand such [^3^H]NMS or [^3^H]QNB to the allosteric site of the M_2_ receptor is negligible at the low concentrations used in the present investigation^3^. The parameters *K*_L_ and *K*_A_ are the equilibrium dissociation constants of L and A for the ortho- and allosteric sites, respectively, of the vacant receptor (*i.e.*, *K*_L_ = [R][L]/[RL], *K*_A_ = [A][R]/[AR]); *K*_Ao_ is the equilibrium dissociation constant of A for the orthosteric site of the vacant receptor (*K*_Ao_ = [R][A]/[RA]).

Either ligand may bind to a receptor occupied by the other to form the ternary complex ARL, as shown in Scheme 2**A**. The reciprocal and equal effect of one ligand on the affinity of the other is represented by the cooperativity factor *α*, in accord with the principle of microscopic reversibility (*i.e.*, *α* = ([AR][RL])/([ARL][R])), and three constants therefore are sufficient to define the system. In a restricted version of the model (Scheme 2**B**), binding of L to the orthosteric site is blocked by the allosteric ligand. Because of the thermodynamic constraint represented by *α*, the two versions of Scheme S2 are indistinguishable at equilibrium.

Binding of the allosteric ligand to the orthosteric site leads to the ternary complex ARA, as shown in Scheme 2**C**. The reciprocal effect on affinity is represented by the homotropic cooperativity factor *β*.

Scheme S2





In Schemes 2**A** and 2**B**, total specific binding of the orthosteric ligand in the presence of the allosteric ligand at thermodynamic equilibrium is given by Equation S7. The concentration of L that achieves half-maximal occupancy of the receptor in the presence of A can be taken as the apparent affinity (*K*_app_), which was formulated as shown in Equation S8 for analyses described in the parent text.

$\text{[RL] + [ARL] = }\frac{[\text{R]}_{\text{t}}\text{[L}]}{K_{\text{L}}\left\{\frac{\left(\text{1+}\frac{\text{[A]}}{K_{\text{A}}}\right)}{\left(\text{1+}\frac{\text{[A]}}{\alpha K_{\text{A}}}\right)}\right\}+[\text{L}]}$ (S7)

$\log K_{\text{app}}=\log\left\{\frac{10^{\log K_{\text{L}}}\left(1+\frac{10^{\log[\text{A}]}}{10^{\log K_{\text{A}}}}\right)}{\left(1+\frac{10^{\log[\text{A}]}}{10^{\log(\alpha K_{\text{A}})}}\right)}\right\}$ (S8)

In Scheme 2**C**, total specific binding of the orthosteric ligand in the presence of the allosteric ligand at thermodynamic equilibrium is given by Equation S9.

$\text{[RL] + [ARL] = }\frac{[\text{R]}_{\text{t}}\text{[L}]}{K_{\text{L}}\left\{\frac{\left(\text{1+}\frac{\text{[A]}}{K_{\text{A}}}+\frac{[\text{A]}}{K_{\text{Ao}}}\left(1+\frac{[\text{A]}}{\beta K_{\text{Ao}}}\right)\right)}{\left(\text{1+}\frac{\text{[A]}}{\alpha K_{\text{A}}}\right)}\right\}+[\text{L}]}$ (S9)

Equations S7 and S9 are rectangular hyperbolic with respect to the concentration of the orthosteric ligand when the concentration of the allosteric ligand is constant. The functional form therefore is unchanged in the presence of the allosteric ligand, which is without effect on the capacity of the receptor for the orthosteric ligand.

Scheme S3 is a kinetic representation of Scheme S2 (**A** and **B**) in which the equilibrium constants of the latter have been replaced by their constituent rate constants. Calculations were performed as described above for Scheme S1, with the values of the rate constraints constrained in an analogous manner (cf. Equations S5 and S6). The accompanying diagrams depict possible arrangements of the two ligands: that is, either ligand can bind and dissociate in the presence of the other (**A**), and access to and from the orthosteric site is blocked by the allosteric ligand (**B**). A blockade could arise from steric hindrance, as depicted in the diagram (**B**), from ionic interactions between the two ligands, or through conformational changes in the receptor.


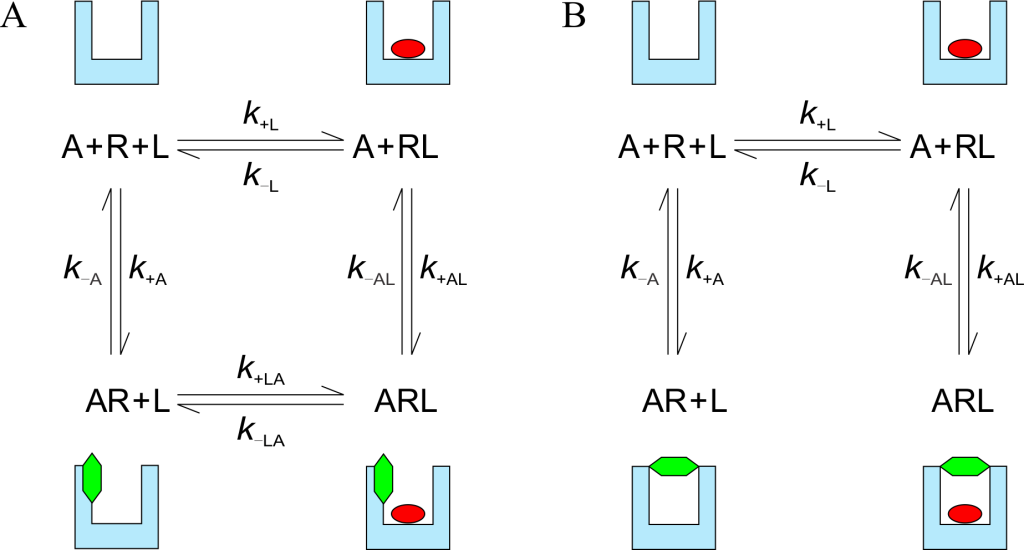
Scheme S3

**Section S2: Binding of orthosteric ligands and the allosteric effects of tacrine**

**Table s1. Parametric values for the effect of tacrine on the binding of [^3^HNMS and [^3^H]QNB to solubilized M_2_ receptor from *Sf*9 cells.**

| Probe | log [T] | | log *K* | *n*_H_ |  | *B*_max_ (nM) |
| --- | --- | --- | --- | --- | --- | --- |
|  |  |  |  |  |  |  |
| [^3^H]QNB*^a^* | *c* | (5) | −9.18 ± 0.13 | 1.01 ± 0.01 |  |  |
|  | −5.0 | (5) | −8.91 ± 0.16 | 1.00 ± 0.03 |  | 0.48 ± 0.06*^d^* |
|  | −4.0 | (3) | −8.48 ± 0.12 | 0.98 ± 0.06 |  |  |
|  | −3.5 | (5) | −8.11 ± 0.14 | 1.01 ± 0.06 |  |  |
|  | −2.5 | (4) | −7.38 ± 0.05 | 1.04 ± 0.03 |  | 0.28 ± 0.04 |
|  |  |  |  |  |  |  |
| [^3^H]NMS*^b^* | *c* | (4) | −8.08 ± 0.02 | 1.02 ± 0.01 |  |  |
|  | −5.0 | (4) | −7.86 ± 0.04 | 0.99 ± 0.02 |  | 0.31 ± 0.03*^d^* |
|  | −3.5 | (4) | −7.35 ± 0.03 | 0.98 ± 0.08 |  |  |
|  | −2.5 | (3) | −6.91 ± 0.02 | 1.02 ± 0.08 |  | 0.18 ± 0.04 |
|  |  |  |  |  |  |  |

The data represented in Figures 1A and 1B were analyzed in terms of Equation 2 (*n* = 1) to obtain the fitted curves shown in the figures and the parametric values listed above. The number of sets of data at each concentration of tacrine (T) is shown in parentheses. Data represented in the table are from 5 experiments with [^3^H]QNB and 4 experiments with [^3^H]NMS. Each measurement was performed in triplicate. Each experiment included one curve in the absence of tacrine and 3–4 curves in the presence of tacrine at the concentrations shown above. *^a^*Figure 1A. *^b^*Figure 1B. *^c^*Tacrine was absent from the assay. *^d^*The value of *B*_max_ was indistinguishable among the different sets of data (*P* > 0.05), which therefore shared the single value shown in the table.

**Table S2. Parametric values for the association and dissociation of [^3^H]QNB at solubilized M_2_ receptor from *Sf*9 cells.**

| **Analysis** | **[^3^H]QNB**  **(nM)** *^a^* | **Protomer** | ***k_+j_**μ*M^−1^min^−1^** | ***k_−j_* min^−1^** |  | ***α*** *^b^* | ***n*, *m*** *^c^* | **log *K_j_*** *^d^* |
| --- | --- | --- | --- | --- | --- | --- | --- | --- |
|  |  |  |  |  |  |  |  |  |
| 1 | 0.316 | R^1^ | 13.7 ± 3.0 | 0.0083 ± 0.0054 | } | 0.020 ± 0.013 | 2.00 | −9.22 |
|  |  | R^2^ | 244 ± 13 | 0.38 ± 0.08 |  |  | 1.00 | −8.81 |
| 2 | 1.00 | R^1^ | 20.6 ± 2.9 | 0.0083 ± 0.0066 | } | 0.027 ± 0.004 | 2.00 | −9.40 |
|  |  | R^2^ | 200 ± 13 | 0.39 ± 0.05 |  |  | 1.00 | −8.72 |
| 3 | 5.62 | R^1^ | 13.2 ± 2.1 | 0.0084 ± 0.0048 | } | 0.070 ± 0.014 | 2.00 | −9.20 |
|  |  | R^2^ | 138 ± 13 | 0.39 ± 0.08 |  |  | 1.00 | −8.56 |
|  |  |  |  |  |  |  |  |  |
| 4 | all*^e^* | R^1^ | 16.0 ± 1.2 | 0.0083 ± 0.0034 | } | 0.043 ± 0.008 | 2.00 | −9.27 |
|  |  | R^2^ | 180 ± 11 | 0.38 ± 0.08 |  |  | 1.00 | −8.67 |
|  |  |  |  |  |  |  |  |  |

The data represented in Figure 1C were combined with data on the dissociation of [^3^H]QNB and analyzed simultaneously in terms of Scheme S1 (Section S1A). Each curve was measured in triplicate, and all samples were from the same preparation of receptor. Data on the association at different concentrations of [^3^H]QNB were analyzed separately (Analyses 1–3) or together (Analysis 4) to obtain the parametric values listed in the table. The fitted curves are shown in Figure 1C. The total concentration of receptor was 400 ± 10 pM, as estimated independently from the binding of [^3^H]QNB at a saturating concentration of 32 nM (*N* = 3), and the value of [R]_t_ in Scheme S1 was fixed accordingly during the fitting procedure. *^a^*The association and dissociation of [^3^H]QNB were measured at the concentrations shown in the table. The dissociation was mono-exponential and occurred at the same rate under all conditions (Eq. 1b, *k*_obsd_); accordingly, the data acquired with receptor labeled at 1 nM [^3^H]QNB were used to define the values of *k*_−_*_j_* in all analyses with Scheme S1.  *^b^*There is a significant increase in the sum of squares when the value of *α* is fixed at 1 (*P* < 0.001). *^c^*Each value was defined by a broad minimum in the sum of squares, as determined by mapping, and was fixed as shown during the fitting procedure. *^d^k_–j_*/*k*_+_*_j_*. There is a significant increase in the sum of squares when the fit is constrained such that *K*_1_ = *K*_2_ (*P* < 0.0001). *^e^*Single values of *k*_±_*_j_* and *α* were common to the 12 sets of data included in the analysis. The fit therefore was mechanistically consistent.


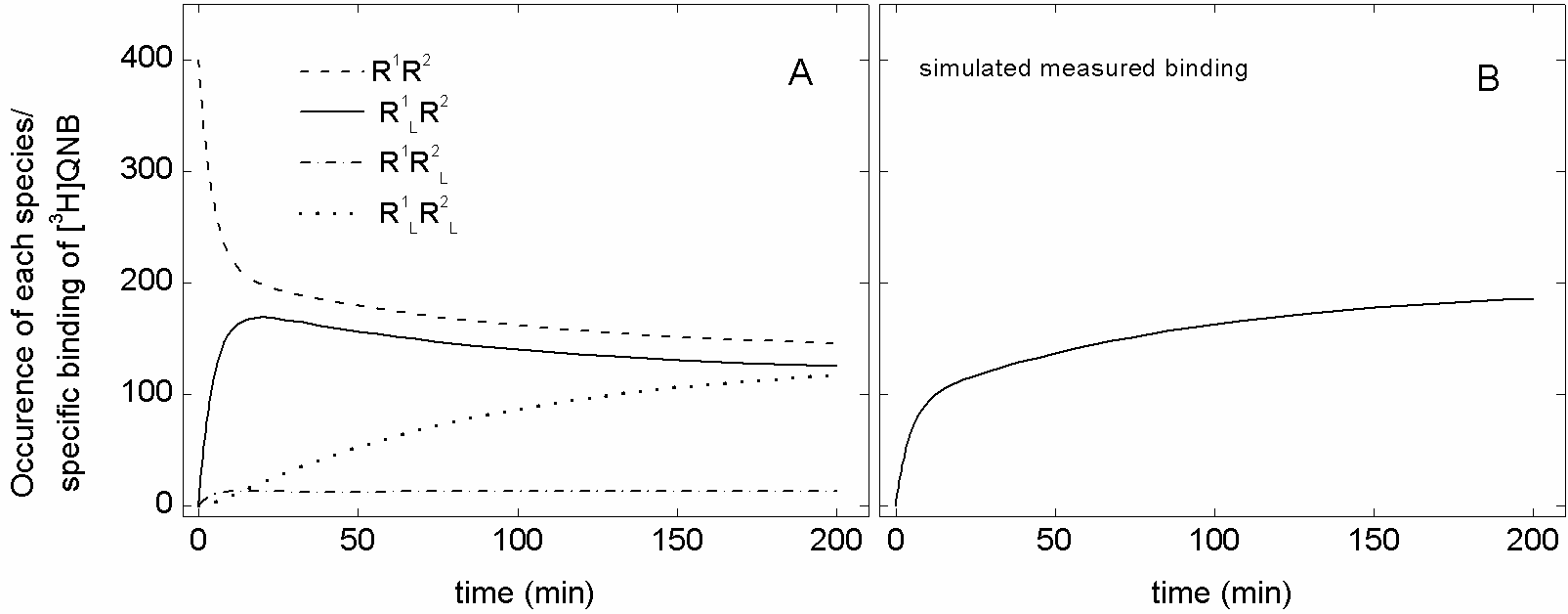


**Figure S2. Contribution of the constituent species to total specific binding in the association of [^3^H]QNB with M_2_ receptor from *Sf*9 cells.** Association and dissociation were measured at a radioligand concentration of 1.0 nM (Fig. 1C), and the data were analyzed in terms of Scheme S1 to obtain the fitted parametric values listed in Table S2 (Analysis 2). Those values were used in turn to calculate the time-dependent appearance of individual species (*i.e.*, $\text{[R}^{1}\text{R}^{2}\text{],}$ $\text{[R}_{\text{L}}^{1}\text{R}^{2}\text{],}$ $\text{[R}^{1}\text{R}_{\text{L}}^{2}\text{],}$ or $\text{[R}_{\text{L}}^{1}\text{R}_{\text{L}}^{2}]$) (**A**, pM) and of total specific binding (*i.e.*,$\text{[R}_{\text{L}}^{1}\text{R}^{2}\text{]}$ + $\text{[R}^{1}\text{R}_{\text{L}}^{2}\text{]}$ + $\text{2[R}_{\text{L}}^{1}\text{R}_{\text{L}}^{2}]$) (**B**, pM). Under the conditions of the reaction, a rapid formation of $\text{R}_{\text{L}}^{1}\text{R}^{2}$is followed by a slower accumulation of $\text{R}_{\text{L}}^{1}\text{R}_{\text{L}}^{2}.$ The amount of $\text{R}^{1}\text{R}_{\text{L}}^{2}$is small throughout.


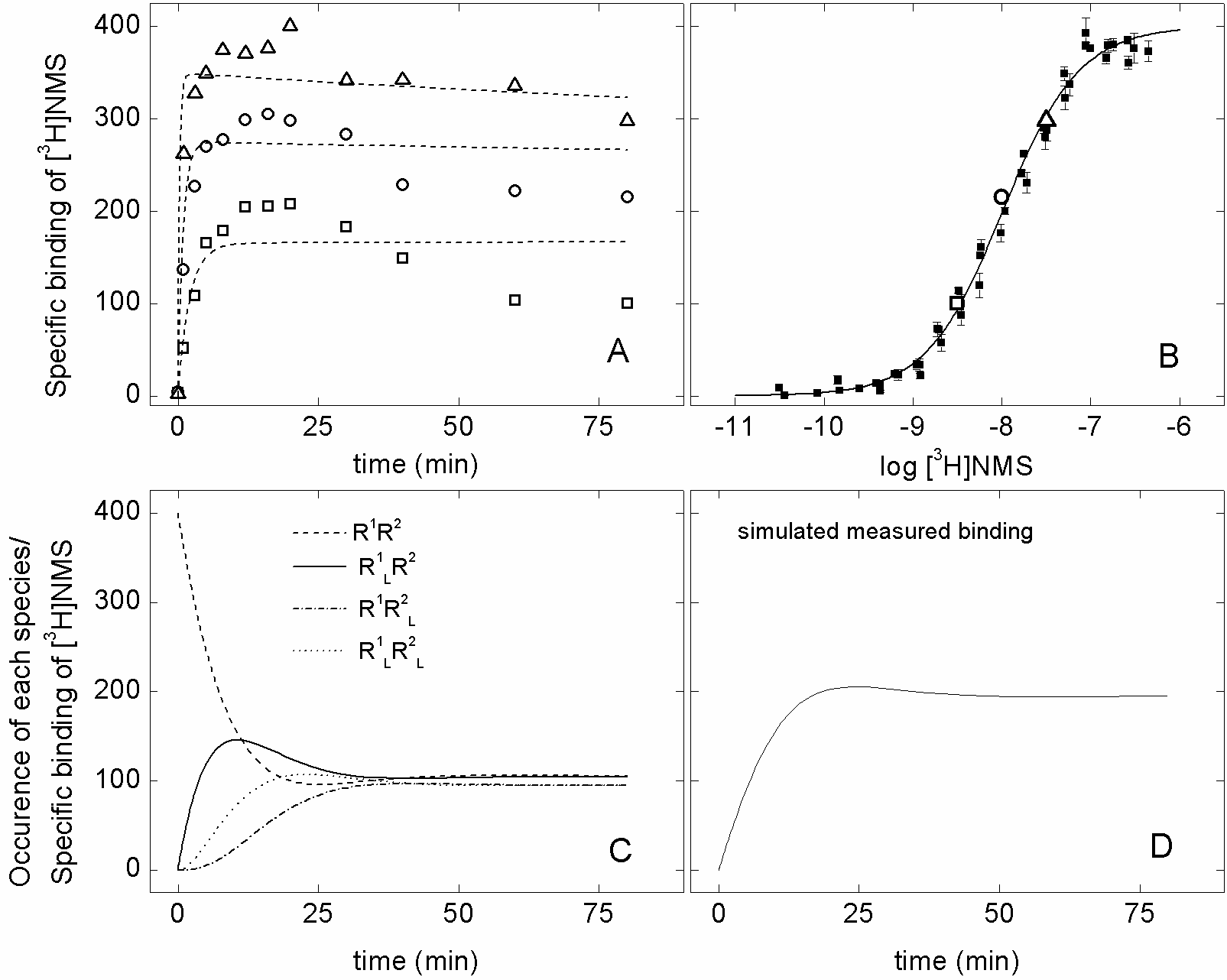


**Figure S3. Binding of [^3^H]NMS to solubilized M_2_ receptor from *Sf*9 cells.** (**A**) Association. Receptor was mixed with [^3^H]NMS (□, 3.16 nM, ○, 10 nM, △ 31.6 nM), and the mixture was incubated at 30 °C for the times shown on the abscissa. Three experiments were carried out at each concentration of [^3^H]NMS, and the estimates of specific binding at the same time and concentration of the radioligand were averaged to obtain the means plotted in the figure (s.e.m./$\bar{x}$ < 0.05). The data represented in the figure were combined with data on the dissociation of [^3^H]NMS from receptor pretreated with the radioligand at a concentration of 10 nM (*N* = 3), and the complete set was analyzed according to Scheme S1 to obtain the broken lines shown in the figure. The total concentration of receptor was 400 pM, as estimated independently from the binding of [^3^H]NMS at a saturating concentration of 1 µM (*N* = 3), and the value of [R]_t_ in Scheme S1 was fixed accordingly during the fitting procedure. Single values of the rate constants and the co-operativity factor *α* were common to all the data, and the fitted parametric values are listed in Table S3. (**B**) Binding at equilibrium. The receptor was mixed with [^3^H]NMS at the concentrations shown on the abscissa, and binding was measured following equilibration of the samples for 15 h at 30° C (closed symbols). The line depicts the best fit of Equation 2 (*n* = 1), and the fitted parametric values are: log *K* = −8.01 ± 0.02 and *n*_H_ = 1.01 ± 0.01. The open symbols are copied from Panel **A** and show the level of binding after incubation for 80 min. (**C, D**) The lines were calculated according to Scheme S1 with the parametric values listed in Table S4. The contributions of individual species are shown in Panel **C** (*i.e.*, $\text{[R}^{1}\text{R}^{2}\text{],}$ $\text{[R}_{\text{L}}^{1}\text{R}^{2}\text{],}$ $\text{[R}^{1}\text{R}_{\text{L}}^{2}\text{],}$ or $\text{[R}_{\text{L}}^{1}\text{R}_{\text{L}}^{2}]$, pM), and total specific binding is shown in Panel **D** (*i.e.*,$\text{[R}_{\text{L}}^{1}\text{R}^{2}\text{]}$ + $\text{[R}^{1}\text{R}_{\text{L}}^{2}\text{]}$ + $\text{2[R}_{\text{L}}^{1}\text{R}_{\text{L}}^{2}]$, pM).

**Table S3. Parametric values for the association and dissociation of [^3^H]NMS at solubilized M_2_ receptor from *Sf*9 cells.**

| **Protomer** | ***k_+j_*(M−^1^min^−1^)** | ***k_−j_* (min^−1^)** |  | ***α*** | ***n*, *m*** | **log *K^a^*** |
| --- | --- | --- | --- | --- | --- | --- |
|  |  |  |  |  |  |  |
| R^1^ | 1.0 × 10^8^ ± 0.85 × 10^8^ | 0.46 ± 0.51 | } | 0.91 ± 0.92 | 1.82 ± 27.7 | −8.33 |
| R^2^ | 1.1 × 10^2^ ± 4.9 × 10^4^ | 0.01 ± 0.05 |  |  | 2.45 ± 40.6 | −3.92 |
|  |  |  |  |  |  |  |

The data represented in Figure S3A were combined with data on the dissociation of [^3^H]NMS from receptor pre-equilibrated with the radioligand at a concentration of 10 nM. Each curve was measured in triplicate, and all samples were from the same preparation of receptor. The combined data were analyzed simultaneously in terms of Scheme S1 to obtain the parametric values listed in the table and the fitted curves shown in Figure S3A. Single values of *k*_±_*_j_* and *α* were common to the 12 sets of data included in the analysis. *^a^k_–j_*/*k*_+_*_j_*.

**Table S4. Parametric values for the simulated association of [^3^H]NMS according to Scheme S1**

| **Species** | ***k_+j_*(M−^1^min^−1^)** | ***k_−j_* (min^−1^)** |  | ***α*** | ***n, m*** | **log *K*** |
| --- | --- | --- | --- | --- | --- | --- |
|  |  |  |  |  |  |  |
| R^1^ | 1.0 × 10^7^ | 0.01 | } | 1 | 1 | −9 |
| R^2^ | 1.0 × 10^2^ | 0.1 |  |  | 1 | −3 |

The values listed in the table were used to simulate the data shown in Figure S3C.

**
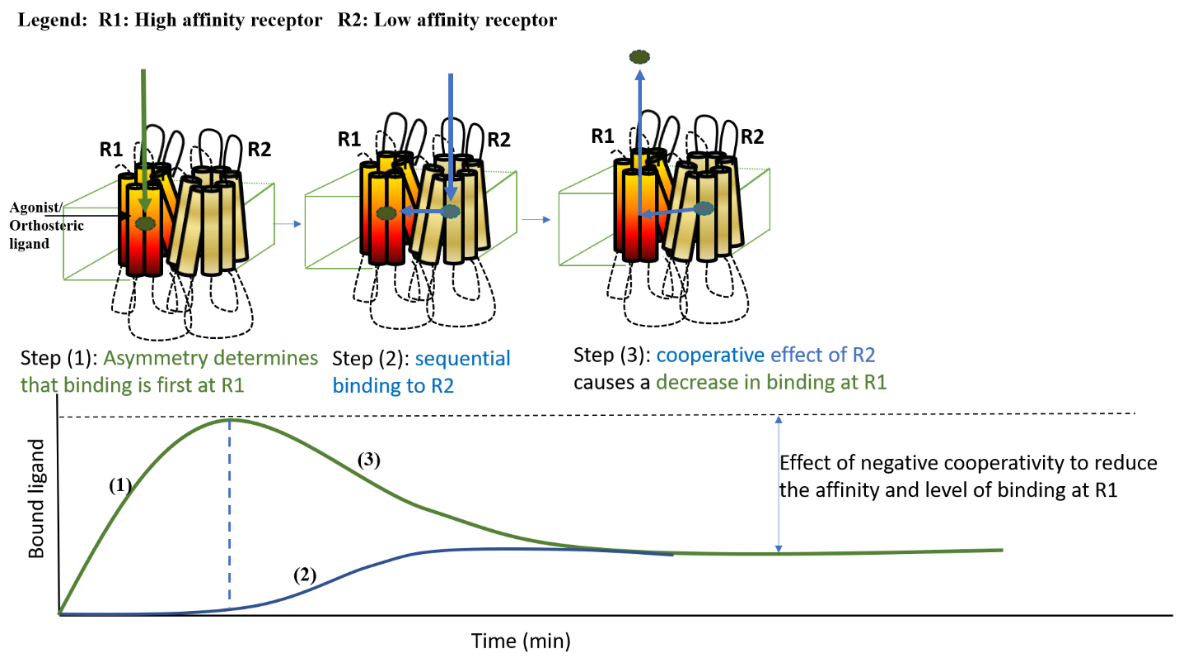
**

**Figure S4. Kinetically determined overshoot in the association of a ligand with a dimer.** Asymmetry and cooperativity working in concert can produce a transient peak in the time-course of binding, as illustrated in the cartoon (*upper panel*) and accompanying sketch (*lower panel*) shown above. The biphasic, green curve in the lower panel depicts the binding of a ligand to R^1^, and the blue curve depicts binding to R^2^; in each case, the amplitude is arbitrary. The process can be viewed as occurring in three steps. First, one equivalent of the ligand binds to the higher-affinity protomer (R^1^) of an asymmetric oligomer (upper panel, green arrow; lower panel, green line). Second, binding of a second equivalent of the ligand to the neighboring protomer (R^2^) is negatively cooperative with respect to that of the first equivalent, which reduces the affinity of the latter for R^1^ (upper panel, blue arrow; lower panel, blue line). Third, the negatively cooperative interaction between ligands at R^1^ and R^2^ leads to a reduction in the amount of bound ligand as the system equilibrates and approaches a plateau. The difference between peak and plateau is the effect of negative cooperativity.


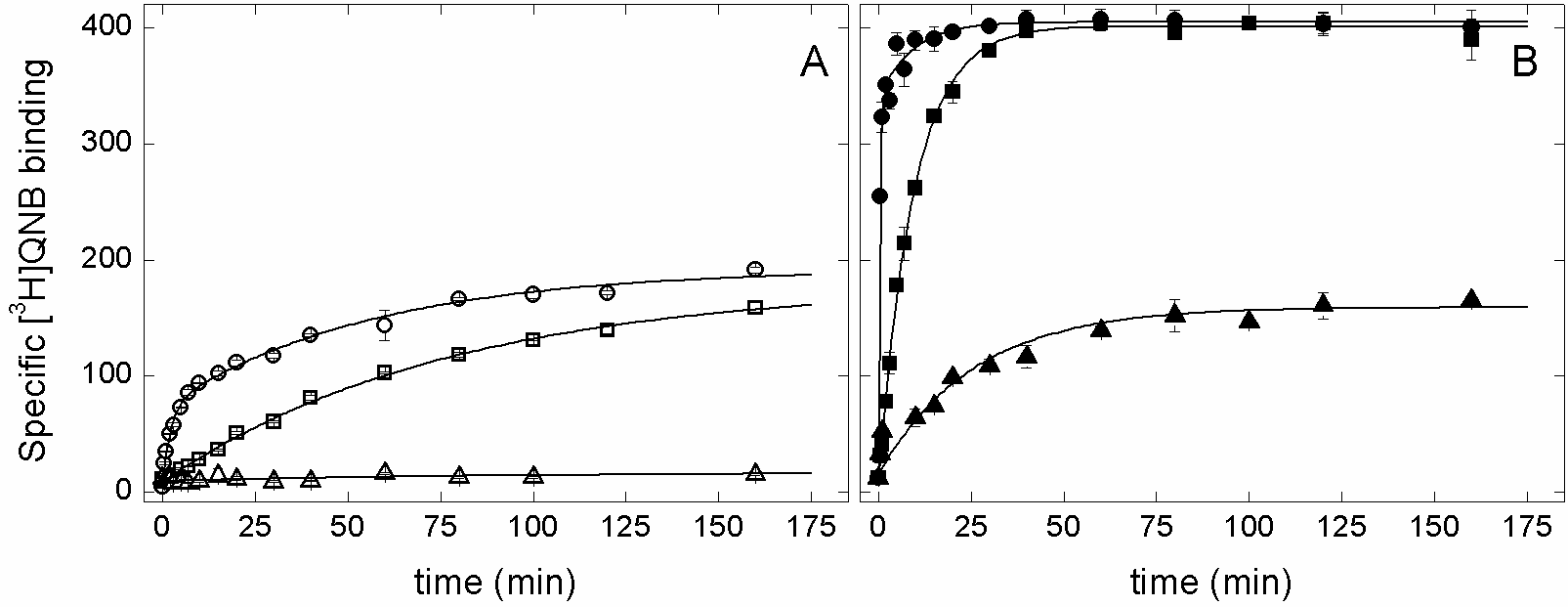


**Figure S5. Effect of tacrine on the association of [^3^H]QNB with solubilized M_2_ receptor from *Sf*9 cells.** Receptor was mixed with [^3^H]QNB (**A**, 1.0 nM; **B**, 17.8 nM) either alone (○, ●) or together with tacrine at a concentration of 10 *μ*M (□, ■) or 316 *μ*M (△, ▲). The mixture was incubated at 30 °C, and binding was measured at the times shown on the abscissa. The lines depict the best fit of Equation 1a to all of the data at each concentration of the radioligand (*N* = 3 or 4), and the parametric values are listed in Table S5.


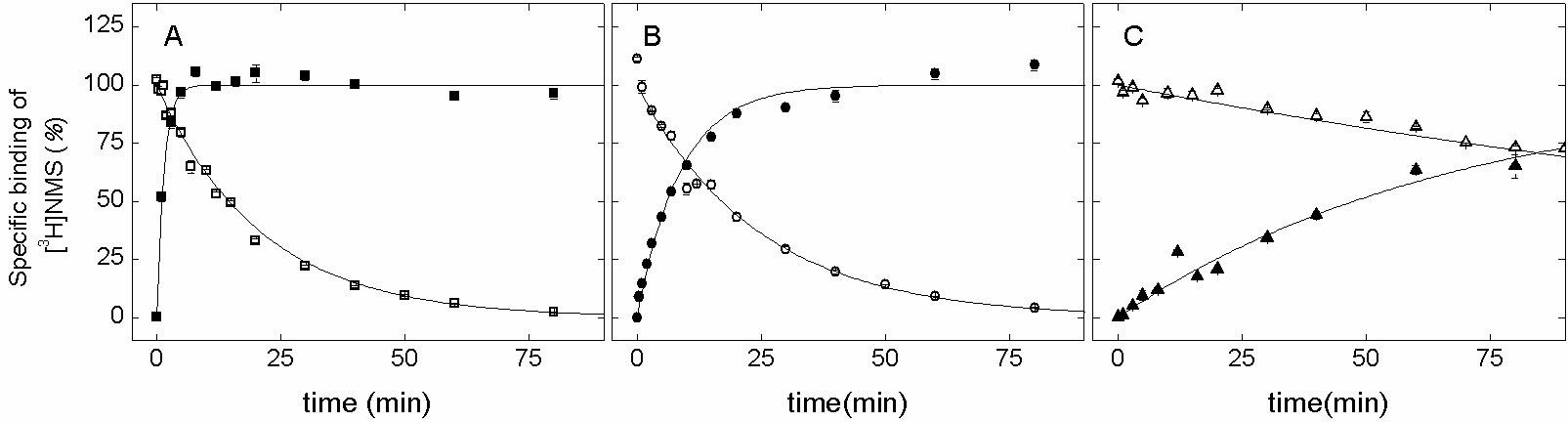


**Figure S6. Effect of tacrine on the association and dissociation of [^3^H]NMS at solubilized M_2_ receptor from *Sf*9 cells.** Association (closed symbols): Receptor was mixed with [^3^H]NMS (10 nM) plus tacrine at a concentration of 1 *μ*M (**A**, 🞏, ■), 10 *μ*M (**B**. ⭘, ●), or 316 *μ*M (**C**, △, ▲). Dissociation (open symbols): Receptor was incubated with [^3^H]NMS (10 nM) for 45 min at 30 °C, and dissociation was initiated by the addition of atropine at a final concentration of 3.0 *μ*M plus tacrine at the concentrations listed above. The mixture was incubated at 30 °C in each case, and binding was measured at the times shown on the abscissa. The lines depict the best fit of Equation 1a (*n* = 1) or 1b, and the fitted parametric values are listed in Table S5.

**Table S5. Rates of association and dissociation of [^3^H]QNB and [^3^H]NMS with and without tacrine in preparations of solubilized M_2_ receptor from *Sf*9 cells.**

|  |  | **Association**  **(Equation 1a)** | | | |  | **Dissociation**  **(Equation 1b)** | |  |  |
| --- | --- | --- | --- | --- | --- | --- | --- | --- | --- | --- |
| **Probe**  **(P)** | **Tacrine**  **(*µ*M)** | **P***^a^*  **(nM)** | ***n*** | ***k*_obsd(_*_j_*)**  **(min^-1^)** | ***k*_+1_**  **(*μ*M^-1^min^-1^)** |  | **P***^a^*  **(nM)** | ***k*_obsd_**  **(min^−1^)** | **log *K*_(kin)_*^c^*** | **log *K*_(eq)_*^d^*** |
|  |  |  |  |  |  |  |  |  |  |  |
| [^3^H]QNB | 0 | 1.00 | 2 | 0.018 ± 0.002*^b^* | 18.0 |  |  | *e* |  |  |
|  | 10.0 | 1.00 | 1 | 0.012 ± 0.001 | 12.0 |  |  | *e* |  |  |
|  | 316 | 1.00 | 1 | 0.009 ± 0.018 | 9.10 |  |  | *e* |  |  |
|  |  |  |  |  |  |  |  |  |  |  |
| [^3^H]QNB | 0 | 17.8 | 2 | 0.11 ± 0.053*^b^* | 6.21 |  |  | *e* |  |  |
|  | 10.0 | 17.8 | 1 | 0.11 ± 0.003 | 6.23 |  |  | *e* |  |  |
|  | 316 | 17.8 | 1 | 0.041 ± 0.003 | 2.24 |  |  | *e* |  |  |
|  |  |  |  |  |  |  |  |  |  |  |
| [^3^H]NMS | 1.00 | 10.0 | 1 | 0.68 ± 0.081 | 68.2 |  | 10 | 0.047 ± 0.002 | −9.15 | — |
|  | 10.0 | 10.0 | 1 | 0.11 ± 0.007 | 11.2 |  | 10 | 0.040 ± 0.001 | −8.44 | −7.86 ± 0.04 |
|  | 316 | 10.0 | 1 | 0.015 ± 0.001 | 1.46 |  | 10 | 0.004 ± 0.001 | −8.55 | −7.35 ± 0.03 |
|  |  |  |  |  |  |  |  |  |  |  |

The data represented in Figures S5 and S6 were analyzed in terms of Equation 1a (*n* = 1 or 2) or 1b to obtain the fitted curves shown in the figures and the parametric values listed above. A single value of *k*_obsd_ was common to the 3 or 4 sets of data acquired at each concentration of the radioligand. *^a^*The concentration listed for [^3^H]QNB or [^3^H]NMS is the mean from all experiments included in the analysis (s.e.m./*μ* < 0.03). *^b^*The value of *k*_obsd(_*_j_*) shown in the table is for the slower component. The value for the faster component (*j* = 1) and the value of *F*_2_ (*j* = 2) are as follows: at 1.00 nM [^3^H]QNB, 0.46 ± 0.04 min^–1^ and 0.64 ± 0.01; at 17.8 nM [^3^H]QNB, 2.7 ± 0.3 min^–1^ and 0.16 ± 0.02.  *^c^*The equilibrium dissociation constant, determined kinetically as the ratio of the rate constants for dissociation (*k*_obsd_, Eq. 1b) and association (*k*_+1_, Eq. 1a), *^d^*The equilibrium dissociation constant, determined at equilibrium. The values are copied from Table S1. *^e^*Not measured.

**
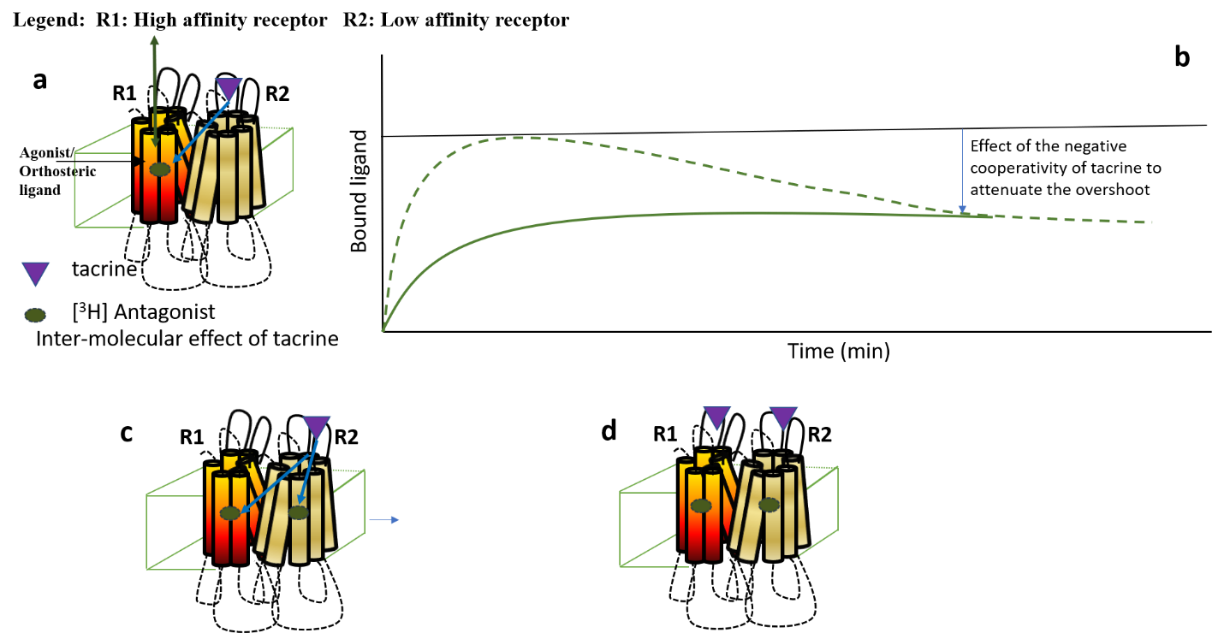
**

**Figure S7. Effect of allosteric ligands on asymmetry and cooperativity.** Tacrine acts to negate the overshoot in the association of orthosteric antagonists. Allosteric ligands (*e.g.*, tacrine) acting at a neighboring protomer would exert a negatively cooperative effect on the asymmetric protomer of higher affinity (**A).** Binding to that site (**B**, broken line) therefore is reduced to that for a site of lower affinity (**B**, solid line). Other modes of association then follow, such as the binding of a second equivalent of orthosteric ligand (**C**) and then a second equivalent of allosteric ligand (*e.g.*, tacrine) (**D**).


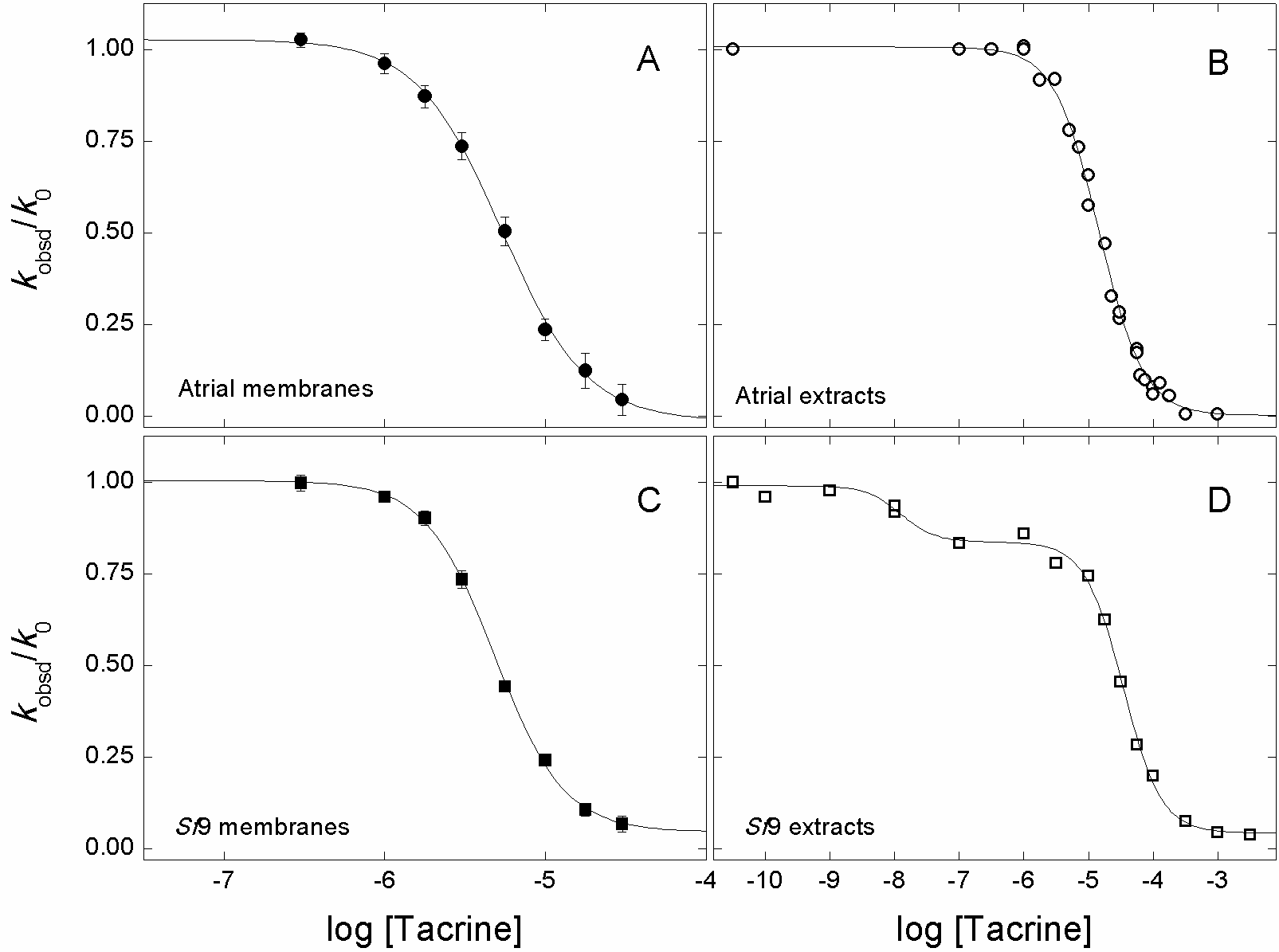


**Figure S8. Effect of tacrine on the rate of dissociation of [^3^H]NMS.** The dissociation of [^3^H]NMS from M_2_ receptor in membranes (**A,** C) and solubilized preparations (**B, D**) from porcine atria (**A** B) and *Sf*9 ells (**C, D**) was followed over time at different concentrations of tacrine. The concentration of [^3^H]NMS was 1.0 nM and 10.0 nM for membranes and extracts, respectively, and the decay was mono-exponential throughout. Each trace was analyzed in terms of Equation 1b, and the rate constant (*k*_obsd_) was normalized to that measured in the absence of tacrine in the same experiment (*k*_0_) to obtain the ratio (*k*_obsd_/*k*_0_) plotted in the figure. The data were analyzed in terms of Equation 3 (*n* = 1 or 2) to obtain the fitted curves shown in the figure and the parametric values listed in Table S6. The Hill coefficient exceeded 1 under all conditions (Table S6), suggesting that tacrine slows the dissociation of [^3^H]NMS via at least two allosteric sites to which it binds in a positively cooperative manner. The biphasic pattern observed with extracts of *Sf*9 cells is suggestive of at least three and perhaps four allosteric sites.

**Table s6. Parametric values for the effect of tacrine on the rate of dissociation of [^3^H]NMS.**

|  |  |  | **[^3^H]NMS**  **(nM)*^a^*** |  |  |  |  | ***k*_obsd_/*k*_0_** | |
| --- | --- | --- | --- | --- | --- | --- | --- | --- | --- |
| **Preparation** | | |  | **log *K*_1_** | **log *K*_2_­** | ***n*_H(1)_** | ***n*_H(2)_** | **[T] = 0** | **[T] → ∞** |
|  |  |  |  |  |  |  |  |  |  |
| Atria | M | (3) | 1.00 | −5.26 ± 0.05 | *b* | 1.65*^d^* ± 0.23 | — | 1.03 ± 0.03 | 0.00 ± 0.05 |
| Atria | S | (6) | 10.0 | −4.84 ± 0.03 | *b* | 1.26*^d^* ± 0.10 | — | 1.00 ± 0.02 | 0.00 ± 0.02 |
| *Sf*9 cells | M | (3) | 1.00 | −5.32 ± 0.02 | *b* | 2.00*^d^* ± 0.14 | — | 1.00 ± 0.01 | 0.05 ± 0.02 |
| *Sf*9 cells | S | (4) | 10.0 | −7.91 ± 0.17 | −4.47 ± 0.03*^c^* | 1.64 ± 2.31 | 1.48*^d^* ± 0.12 | 0.99 ± 0.01 | 0.04 ± 0.01 |
|  |  |  |  |  |  |  |  |  |  |

The dissociation of [^3^H]NMS from membrane-bound (M) and solubilized (S) M_2_ receptor was followed at different concentrations of tacrine ([T]) in preparations from porcine atria and *Sf*9 cells. The dependence of the normalized rate constant (*k*_obsd_/*k*_0_) on log [T] was analyzed in terms of Equation 3 (*n* = 1 or 2) to obtain the fitted curves shown in Figure S8 and the parametric values listed above. Values in parentheses indicate the total number of experiments, where each experiment included measurements at several concentrations of tacrine (T) and a control in its absence. *^a^*The concentration of [^3^H]NMS is the mean from all experiments included in the analysis (s.e.m./*μ* < 0.02). *^b^*One term is sufficient to describe the data (*i.e.*, *n* = 1 in Equation 3). *^c^*Two terms are required to describe the data (*n* = 2), and the fitted value of *F*_2_ is 0.84 ± 0.02. *^d^*The value significantly exceeds 1 (*P* < 0.01).

**Table S7. Parametric values for the effect of tacrine on the binding of [^3^H]MS to solubilized M_2_ receptor from porcine atria and *Sf*9 cells. Effect of temperature and the time of incubation.**

| **Preparation** | **Time of**  **incubation** |  | **log *K*_1_** | | **log *K*_2_­** |  | ***n*_H(1)_** | ***n*_H(2)_** | | | ***F*_2_** |
| --- | --- | --- | --- | --- | --- | --- | --- | --- | --- | --- | --- |
|  |  |  |  | |  |  |  |  | | |  |
| Atria, 4 ºC*^a^* | 7 d (4) |  | −5.61 ± 0.03 | | −4.24 ± 0.09 |  | 2.27*^e^* ± 0.32 | 0.94 ± 0.07 | | | 0.64 ± 0.05 |
|  | 14 d (3) |  |  |  | −3.54 ± 0.05 |  |  |  |  |  | 0.54 ± 0.02 |
|  |  |  |  | |  |  |  |  | | |  |
| Atria, 30 ºC*^b^* | 3 h (4) |  | −5.14 ± 0.01 | | −4.05 ± 0.08 |  | 1.60*^e^* ± 0.06 | 1.36*^e^* ± 0.11 | | | 0.33 ± 0.04 |
|  | 6 h (3) |  |  |  | −3.69 ± 0.07 |  |  |  |  |  | 0.35 ± 0.02 |
|  | 9 h (4) |  |  |  | −3.53 ± 0.08 |  |  |  |  |  | 0.30 ± 0.02 |
|  | 15 h (6) |  |  |  | −3.26 ± 0.06 |  |  |  |  |  | 0.30 ± 0.02 |
|  | 21 h (5) |  |  |  | −3.04 ± 0.08 |  |  |  |  |  | 0.26 ± 0.01 |
|  | 30 h (4) |  |  |  | −3.04 ± 0.05 |  |  |  |  |  | 0.28 ± 0.01 |
|  |  |  |  | |  |  |  |  | | |  |
| Atria, 37 ºC*^c^* | 9 h (3) |  | −5.07 ± 0.06 | | −3.51 ± 0.12 |  | 1.33*^e^* ± 0.23 | 1.27 ± 0.17 | | | 0.39 ± 0.08 |
|  | 15 h (3) |  |  |  | −3.10 ± 0.11 |  |  |  |  |  | 0.40 ± 0.07 |
|  | 21 h (4) |  |  |  | −3.07 ± 0.07 |  |  |  |  |  | 0.40 ± 0.07 |
|  |  |  |  | |  |  |  |  | | |  |
| *Sf*9 cells, 30 ºC*^d^* | 9 h (4) |  | −4.85 ± 0.07 | | −3.82 ± 0.31 |  | 1.72*^e^* ± 0.32 | 0.93 ± 0.22 | | | 0.66 ± 0.18 |
|  | 15 h (4) |  |  |  | −3.40 ± 0.12 |  |  | 1.22 ± 0.24 | | | 0.44 ± 0.10 |
|  | 21 h (3) |  |  |  | −3.32 ± 0.12 |  |  | 1.16 ± 0.55 | | | 0.41 ± 0.16 |
|  | 30 h (3) |  |  |  | −3.21 ± 0.22 |  |  | 0.90 ± 0.47 | | | 0.58 ± 0.21 |
|  |  |  |  |  | |  |  | |  |  | |

The data represented in Figures 2 and S9 were analyzed according to Equation 3 (*n* = 2) to obtain the fitted curves shown in the figures and the parametric values listed above. Data acquired from the same preparation at the same temperature were analyzed in concert. Single values of log *K*_1_, *n*_H(1)_, and *n*_H(2)_ were shared as indicated by the brackets, and those constraints were without significant effect on the sum of squares (*P* > 0.5). The number of independent experiments is shown in parentheses. *^a^*Figure 2G. *^b^*Figure 2A–F. *^c^*Figure 2D–E. *^d^*Figure S9. *^e^* The value significantly exceeds 1 (*P* < 0.01).

**Table S8. Parametric values for the effect of tacrine on the binding of [^3^H]NMS to M_2_ receptor in membranes of *Sf*9 cells. Effect of the order of mixing.**

|  | **Time***^a^* | |  | **log *K*_1_** | | **log *K*_2_­** |  | ***n*_H(1)_** | ***n*_H(2)_** | ***F*_1_** | ***F*_2_** |
| --- | --- | --- | --- | --- | --- | --- | --- | --- | --- | --- | --- |
|  |  |  |  |  | |  |  |  |  |  |  |
| Simultaneous addition | 3 h | (3) |  | −6.15 ± 0.04 | | −4.87 ± 0.14 |  | 1.67*^d^* ± 0.19 | 2.28 ± 1.31 | 0.78 | 0.22 ± 0.05 |
|  | 16 h | (4) |  |  |  | −4.04 ± 0.14 |  |  |  | 0.80 | 0.20 ± 0.03 |
|  |  |  |  |  | |  |  |  |  |  |  |
| Premix with [^3^H]NMS | 3 h | (3) |  | −6.17± 0.08 | | −4.60 ± 0.04 |  | 1.19 ± 0.11 | 2.48 ± 0.78 | 3.54 | −2.54 ± 0.37 |
|  | 16 h | (4) |  |  |  | −4.05 ± 0.17 |  |  | 1.85 ± 2.15 | 1.32 | −0.32 ± 0.09 |
|  |  |  |  |  |  | |  |  |  |  |  |

The data represented in Figure 2H–I were analyzed in terms of Equation 3 (*n* = 2) to obtain the fitted curves shown in the figure and the parametric values listed above. Data obtained following the same order of mixing were analyzed in concert. Single values of log *K*_1_ and *n*_H(_*_j_*) were shared as indicated by the brackets, and the constraints were without significant effect on the sum of squares (*P* > 0.05). The number of independent experiments is shown in parentheses. The mean concentration of [^3^H]NMS was 0.30 nM (*N* = 14). *^a^*Time of incubation in the presence of both ligands. *^d^*The value significantly exceeds 1 (*P* = 0.0009).


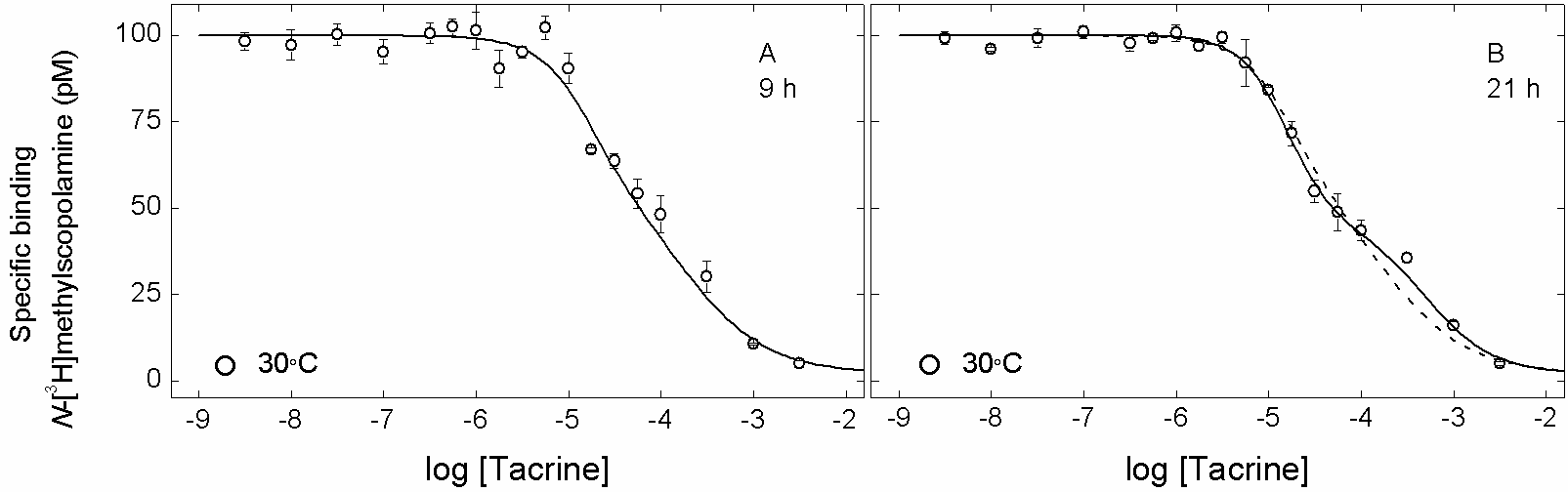


**Figure S9. Equilibration of tacrine and [^3^H]NMS with solubilized M_2_ receptor from *Sf*9 cells.** Tacrine and [^3^H]NMS (10 nM) were mixed simultaneously with aliquots of the extract, and total binding was measured after incubation of the reaction mixture for 9 h (**A**) or 21 h (**B**) at 30 °C. Binding also was measured after incubation for 15 h and 30 h (not shown), and all of the data were analyzed simultaneously according to Equation 3 (*n* = 2). The solid lines in the figure depict the best fit of Equation 3 (*n* = 2) to the corresponding data, and the parametric values for all times of incubation are listed in Table S7. The fitted curve in Panel **A** is reproduced as the dashed line in Panel **B**.

**Table S9. Parametric values for the effect tacrine on the binding of [^3^H]NMS and [^3^H]QNB to solubilized M_2_ receptor from *Sf*9 cells at different concentrations of the radioligand.**

| **Radioligand** | | | |  |  |  |  |  |  |  |
| --- | --- | --- | --- | --- | --- | --- | --- | --- | --- | --- |
|  | **(nM)*^a^*** | **(%)*^b^*** |  |  | **log *K*_1_** | **log *K*_2_­** |  | ***n*_H(1)_** | ***n*_H(2)_** | ***F*_2_** |
|  |  |  |  |  |  |  |  |  |  |  |
| [^3^H]NMS | 3.16 | 24.4 | (3) |  | −5.61 ± 0.10 | −3.91 ± 0.10 |  | 1.33 ± 0.11*^c^* | 0.99 ± 0.07 | 0.43 ± 0.04 |
|  | 5.60 | 36.5 | (3) |  | −5.36 ± 0.08 | −3.62 ± 0.09 |  |  |  | 0.49 ± 0.03 |
|  | 10.1 | 50.6 | (6) |  | −4.79 ± 0.09 | −3.39 ± 0.16 |  |  |  | 0.53 ± 0.07 |
|  | 17.8 | 64.5 | (3) |  | −5.66 ± 0.09 | −3.49 ± 0.05 |  |  |  | 0.60 ± 0.02 |
|  | 31.6 | 76.4 | (3) |  | −4.87 ± 0.10 | −3.24 ± 0.07 |  |  |  | 0.86 ± 0.04 |
|  | 112 | 92.0 | (3) |  | −5.55 ± 0.76 | −3.00 ± 0.05 |  |  |  | 0.95 ± 0.02 |
|  |  |  |  |  |  |  |  |  |  |  |
| [^3^H]QNB | 0.31 | 30.0 | (3) |  | −5.68 ± 0.07 | −3.72 ± 0.11 |  | 1.55 ± 0.33 | 0.89 ± 0.06 | 0.58 ± 0.05 |
|  | 0.560 | 43.1 | (3) |  |  | −4.20 ± 0.12 |  |  |  | 0.60 ± 0.04 |
|  | 1.01 | 57.4 | (3) |  |  | −3.92 ± 0.18 |  |  |  | 0.66 ± 0.09 |
|  | 1.79 | 70.6 | (3) |  |  | −3.77 ± 0.09 |  |  |  | 0.72 ± 0.09 |
|  | 3.17 | 81.0 | (3) |  |  | −3.69 ± 0.07 |  |  |  | 0.75 ± 0.04 |
|  | 10.0 | 93.0 | (3) |  |  | −3.30 ± 0.05 |  |  |  | 0.95 ± 0.03 |
|  |  |  |  |  |  |  |  |  |  |  |

Data acquired at six concentrations of [^3^H]NMS and [^3^H]QNB were analyzed according to Equation 3 (*n* = 2) to obtain the parametric values listed above. The data and fitted curves for three concentrations are illustrated in Figure 3. All data obtained with the same radioligand were analyzed in concert. Single values of log *K*_1_ and *n*_H(_*_j_*) were shared as indicated by the brackets; the constraints were without significant effect on the sum of squares (*P* > 0.2). The number of independent experiments is shown in parentheses.  *^a^*The concentration of the radioligand is the mean from all experiments represented in the analysis ([^3^H]NMS, S.E.M./*μ* < 0.05; [^3^H]QNB, S.E.M./*μ* < 0.05). *^b^*Occupancy of the receptor by the radioligand (P) in the absence of tacrine; the values were calculated as [P]/([P] + *K*), with the value of *K* taken as 10 nM for [^3^H]NMS or 0.74 nM for [^3^H]QNB (Table S1). *^c^*The value significantly exceeds 1 (*P* < 0.001).

**Table S10. Parametric values for the effect of tacrine on the binding of [^3^H]NMS to wild-type and mutant M_2_ receptors in membranes from CHO cells.**

| **Receptor** | **[^3^H]NMS**  **(nM)** | |  | **log *K*_1_** | | **log *K*_2_** | | ***n*_H(1)_** | ***n*_H(2)_** | | ***F*_2_** |
| --- | --- | --- | --- | --- | --- | --- | --- | --- | --- | --- | --- |
|  |  |  |  |  | |  | |  |  | |  |
| Wild-type | 1.0 | (5) |  |  | |  | |  |  | | 0.14 ± 0.06 |
|  | 3.0 | (3) |  | –5.75 ± 0.05 | | –3.88 ± 0.32 | | 1.53 ± 0.25*^a^* | 0.70 ± 0.23 | | 0.35 ± 0.09 |
|  | 10.0 | (3) |  |  | |  | |  |  | | 0.76 ± 0.15 |
|  |  |  |  |  | |  | |  |  | |  |
| Y80A, Y83A | 1.0 | (6) |  |  | |  | |  |  | | 0.13 ± 0.06 |
|  | 3.0 | (4) |  | –5.41 ± 0.06 | | –4.04 ± 0.12 | | 1.0*^b^* | 1.0*^b^* | | 0.41 ± 0.06 |
|  | 10.0 | (4) |  |  | |  | |  |  | | 0.80 ± 0.06 |
|  |  |  |  |  |  | |  | |  |  | |

The data represented in Figures 6B and 6E were analyzed in terms of Equation 3 (*n* = 2) to obtain the fitted curves shown in the figures and the parametric values listed above. Data from the same receptor were analyzed in concert, and single values of log *K_j_* and *n*_H(_*_j_*) were shared as indicated by the brackets. Those constraints were without significant effect on the sum of squares (*P* > 0.3). The number of experiments is shown in parentheses. *^a^*The value significantly exceeds 1 (*P* < 0.01). *^b^*The value was indistinguishable from 1 (*P* > 0.2) and was fixed accordingly during the fitting procedure.

**Section S3: Supplementary Methods**

**Ligands, Detergents, and other Materials.** *N*-[^3^H]Methylscopolamine ([^3^H]NMS) was obtained as the chloride salt from PerkinElmer (lots 3406081 and 3474009, 83.5 Ci/mmol; 3436143, 3499213 and 3538031, 81.0 Ci/mmol) and as the bromide salt from Amersham Biosciences (batches B-32, 84.0 Ci/mmol; B-35, B-36, 81.0 Ci/mmol and B-38, 80 Ci/mmol). (−)-[^3^H]Quinuclidinylbenzilate ([^3^H]QNB) was purchased from PerkinElmer (lots 3363717, 37.0 Ci/mmol, 3467373, 39 Ci/mmol; 3499844, 42.0 Ci/mmol) and Amersham (batches B-49, 49.0 Ci/mmol; B-50, 41 Ci/mmol). Ethanol supplied with the radioligand was removed by evaporation prior to use. Atropine sulfate (Batches 69H0545 and 88H0122) and tacrine hydrochloride (batches 3B/92010–8) were obtained from Tocris.

Digitonin used in the solubilization, purification and characterization of the receptor was obtained from the sources identified previously (Ma *et al*., 2007). Protease inhibitors and other chemicals were from sources identified previously (Ma *et al*., 2007) unless stated otherwise. Polypropylene columns used in binding assays were obtained from Kontes (Disposaflex, 0.8 × 6.5 cm) and were packed with Sephadex G-50 from Sigma-Aldrich. Fiberglass filters used in binding assays were from Whatman Schleicher and Schuell (No. 32). Protein concentration was estimated by means of the BCA Protein Assay Kit from Pierce (Ma *et al*., 2007).

**Muscarinic receptor from porcine atria.** The M_2_ receptor is the predominant muscarinic subtype in porcine atria CHIDIAC. Procedures for the preparation of sarcolemmal membranes and extraction of the receptor have been described in detail previously^4^. Briefly, atria were collected immediately after slaughter, taking care to avoid the sinus and atrioventricular nodes. The tissue was washed twice with ice-cold PBS (20 mM KH_2_PO_4_, 150 mM NaCl, NaOH to pH 7.40) and homogenized in buffer A (20 mM imidazole, 1 mM EDTA, 0.1 mM PMSF, 0.02% NaN_3_, HCl to pH 7.60) supplemented with benzamidine (1 mM), pepstatin A (20 *µ*g/mL), leupeptin (0.2 *µ*g/mL), and bacitracin (200 *µ*g/mL). The resulting homogenate was fractionated by centrifugation on a sucrose density gradient (13–28%), and the sarcolemmal fraction was resuspended in buffer A or buffer B (20 mM HEPES, 1 mM EDTA, 0.1 mM PMSF, NaOH to pH 7.40). Following quantification of the receptor by means of [^3^H]quinuclidinylbenzilate, membranes were collected by centrifugation and stored at −75 ºC until required for extraction in detergent (buffer A) or for binding assays on resuspended samples (buffer B).

To solubilize the receptor, sarcolemmal membranes from sucrose density gradients were thawed and resuspended in buffer A supplemented with digitonin (0.36%) and cholate (0.08%). The concentration of protein was 5.5 g/L. The mixture was incubated for 10 min at 24 ºC and centrifuged for 45 min at 4 ºC and 100,000 × *g*. The resulting pellet was resuspended in buffer A supplemented with digitonin (0.8%) and cholate (0.08%), and the mixture was incubated for a further 10 min, diluted with an equal volume of buffer A, and centrifuged as before. The receptor-containing supernatant was divided into aliquots that were stored at −75 ºC until required for binding assays.

**M_2_­ muscarinic receptor from CHO and *Sf*9 Cells.** CHO cells stably expressing the human M_2_ receptor were maintained at 37 °C, 5% CO_2_, and 100% humidity in F-12 medium (Sigma-Aldrich) supplemented with fetal bovine serum (5%), penicillin (100 units/mL), and streptomycin (100 *μ*g/mL). The cells were harvested and homogenized in ice-cold 5 mM phosphate buffer (1 mM KH_2_PO_4_, 4 mM Na_2_HPO_4_, pH 7.4) with three pulses (15 s) of a Bio Homogenizer (Biospec Products, Inc., Bartlesville, OK), and the mixture was centrifuged for 30 min at 4 ºC and 50,000 × g. The pellet was resuspended in ice-cold 5 mM phosphate buffer, divided into aliquots, and stored at −70 °C. Further details have been described previously^5,6^.

*Sf*9 cells coexpressing human M_2_ receptors tagged at the *N*-terminus with either the c-Myc or the FLAG epitope were prepared as described previously PARK. The cells were cultured at 27 °C in Ex-Cell 400 insect medium (JRH Biosciences) containing fetal bovine serum albumin (2%), Fungizone (1%), and gentamycin (0.1%) (all from Gibco-BRL) and grown to confluence at a density of 2 × 10^6^ cell/mL, when they were infected with one of the baculoviruses or coinfected with equivalent titres of both at a total multiplicity of infection of 5 pfu per cell. Cells were harvested 48 h after infection by centrifugation for 15 min at 4 ºC and 1,000 × *g*, and the pellet was stored at −75 °C.

Harvested *Sf*9 cells were homogenized and washed twice in buffer D (20 mM KH_2_PO_4_, 20 mM NaCl, 1 mM EDTA, 0.1 mM PMSF, Complete Protease Inhibitor tablets, NaOH to pH 7.4) by centrifugation for 45 min at 4 ºC and 100,000 × *g*. Washed membranes destined for binding assays were resuspended in buffer B and divided into aliquots that were centrifuged for 10 min at 4 ºC and 18,000 × *g*. The pellets were stored at −75 ˚C. Receptor was extracted from washed membranes in digitonin–cholate (0.86% digitonin, 0.17% cholate) as described previously^1^. Extract destined for binding assays was divided into aliquots and stored at −75 ºC. Receptor was purified from the solubilized extract by successive passage on DEAE-Sepharose, ABT–Sepharose, and hydroxyapatite as described previously^7^. The final concentrations of digitonin and cholate were 0.1% and 0.02%, respectively. Purified receptor was stored at −75 °C.

**Binding assays.** Assays were performed in buffer B except where stated otherwise. Magnesium-free buffers of comparatively low ionic strength are optimal for detecting the allosteric effects^5^. For studies on detergent-solubilized extracts, buffer B was supplemented with 0.1% digitonin and 0.02% cholate. Ligands to be mixed with the receptor were dissolved in buffer B or other buffer as required for the assay. Measurements were made in triplicate unless stated otherwise.

The rate of dissociation of a radioligand from membrane-bound M_2_ receptor was measured in two-point assays as described previously. Membranes were suspended in buffer D (20 mM KH_2_PO_4_, 20 mM NaCl, 1 mM EDTA, 0.1 mM PMSF, Complete Protease Inhibitor tablets, NaOH to pH 7.4) at a protein concentration of 2–5 *µ*g/mL, and aliquots of the mixture (1 mL) were incubated with [^3^H]NMS for 30 min at 24 °C or with [^3^H]QNB for 60 min at 37 °C. Net dissociation of the radioligand was initiated by the addition of atropine (3 *µ*M in a final volume of 2 mL), either alone or together with tacrine at the required concentration. After further incubation for the specified time, the reaction was terminated by filtration of the sample through fibreglass filters pre-treated with a solution of polyethylenimine (0.1%) in water. The filters then were washed twice with 5 mL of ice-cold phosphate buffer (8 mM KH_2_PO_4_, 32 mM Na_2_HPO_4_, pH 7.4) and assayed for radioactivity as described below.

To measure the rate of dissociation of [^3^H]NMS or [^3^H]QNB from solubilized M_2_ receptor, an aliquot of the radioligand dissolved in buffer B was placed in a polypropylene microcentrifuge tube (2 mL) and mixed with the receptor in the ratio 50:3 (v/v). The radioligand was present at a final concentration near its equilibrium dissociation constant unless stated otherwise ([^3^H]NMS, *K*_D_ = 10 nM; [^3^H]QNB, *K*_D_ = 1 nM). The reaction mixture was incubated for 45 min ([^3^H]NMS) or 2 h ([^3^H]QNB), and three aliquots (50 *μ*L each) were removed to determine the level of initial binding (*i.e*., t = 0). Net dissociation of the radioligand then was initiated by the addition of atropine at a final concentration of 3 *μ*M. Dilution of the receptor at this step was negligible (< 0.02%), and the concentration of atropine was sufficient to block reassociation at all orthosteric sites. Following the addition of atropine, aliquots of the reaction mixture (50 *μ*L) were removed in duplicate at times up to 3 min and in triplicate thereafter. The reaction was terminated by separation of the free and bound radioligand on Sephadex G-50 as described previously.

To measure the rate of association of [^3^H]NMS or [^3^H]QNB with solubilized M_2_ receptor, an aliquot of the latter was added to a polypropylene microcentrifuge tube (2 mL) containing the radioligand or the radioligand and plus tacrine dissolved in buffer B. The ratio of volumes was 50:3 (ligands:receptor), Aliquots of the reaction mixture were removed in duplicate at times up to 2 min and in triplicate thereafter, and the reaction was terminated by separation of the free and bound radioligand on Sephadex G-50. The total concentration of the radioligand was estimated in a sample taken from the reaction mixture, and the total concentration of receptor was estimated independently from the level of binding at a saturating concentration of the radioligand.

Various preparations and conditions were examined for the binding of [^3^H]NMS or [^3^H]QNB at graded concentrations of the radioligand or at fixed concentrations of the radioligand and graded concentrations of tacrine. Aliquots of homogenized membranes from porcine atria or CHO cells (1 mL) or of detergent-solubilized extracts (3 *μ*L) from porcine atria or *Sf*9 cells were mixed with the radioligand alone, the radioligand plus tacrine, or the radioligand plus atropine according to one of two protocols that differed in the order of mixing. The samples were incubated for the required time at the specified temperature, and the free and bound radioligand were separated by filtration on fiberglass filters or by chromatography on Sephadex G-50. The various protocols have been described in detail previously^6^.

To measure the level of radioactivity, each sample was counted twice for 5 min by liquid scintillation spectrometry (Beckman LS6500 or Packard 2100TR). The background was subtracted, and the rate of disintegration (dpm) was determined from the counting efficiency as estimated by means of quenched standards. Individual estimates of dpm from replicate samples counted twice were averaged to obtain the mean and standard error used in subsequent analyses.

**REFERENCES**

1. Ma, A. W., Redka, D. S., Pisterzi, L. F., Angers, S. & Wells, J. W. Recovery of oligomers and cooperativity when monomers of the m(2) muscarinic cholinergic receptor are reconstituted into phospholipid vesicles. *Biochemistry* **46**, 7907–7927 (2007).

2. Park, P., Sum, C. S., Hampson, D. R., Van Tol, H. H. & Wells, J. W. Nature of the oligomers formed by muscarinic m2 acetylcholine receptors in Sf9 cells. *Eur. J. Pharmacol* **421**, 11–22 (2001).

3. Redka, D. S., Heerklotz, H. & Wells, J. W. Efficacy as an intrinsic property of the M(2) muscarinic receptor in its tetrameric state. *Biochemistry* **52**, 7405–7427 (2013).

4. Chidiac, P., Green, M. A., Pawagi, A. B. & Wells, J. W. Cardiac muscarinic receptors. Cooperativity as the basis for multiple states of affinity. *Biochemistry* **36**, 7361–7379 (1997).

5. Shivnaraine, R. V. *et al.* Allosteric modulation in monomers and oligomers of a G protein-coupled receptor. *Elife* **5**, (2016).

6. Shivnaraine, R. V., Huang, X.-P., Seidenberg, M., Ellis, J. & Wells, J. W. Heterotropic cooperativity within and between protomers of an oligomeric M<inf>2</inf> muscarinic receptor. *Biochemistry* **51**, (2012).

7. Redka, D. S. *et al.* Coupling of G proteins to reconstituted monomers and tetramers of the M<inf>2</inf> muscarinic receptor. *J. Biol. Chem.* **289**, (2014).
